# Implementation of an innovative ERAS protocol in cardiac surgery: A qualitative evaluation from patients’ perspective

**DOI:** 10.1371/journal.pone.0303399

**Published:** 2024-05-10

**Authors:** Mona Elisabeth Schmid, Sina Stock, Evaldas Girdauskas

**Affiliations:** Cardiothoracic Surgery, University Hospital Augsburg, Augsburg, Germany; Sinai Health System, CANADA

## Abstract

**Objective:**

Enhanced recovery after surgery (ERAS) protocols aim to optimize the recovery process for patients after surgical interventions and focus on patient-centered care. In cardiac surgery, the ERAS concept is still in its early stages. Our university hospital has implemented an innovative ERAS protocol for minimally invasive heart valve surgery since 2021. Therefore, our study aimed to comprehensively assess the patient experience within this newly established ERAS protocol and focused on exploring and understanding the nuances of optimal healthcare delivery under the ERAS framework from the unique perspective of the patients undergoing cardiac surgery.

**Methods:**

Qualitative research was conducted using semi-structured interviews. Data was analyzed using Kuckartz´s qualitative content analysis.

**Results:**

The following main themes emerged from the 12 completed patient interviews: 1) information and communication flow, 2) perioperative patient care, and 3) rehabilitation. Patients found the pre-operative patient education and preconditioning very helpful. Patients were satisfied with the flow of information throughout the whole perioperative care process. Most patients expressed a need for more information about the course of surgery. The intensity of care provided by different professions was perceived as optimal. The support and inclusion of relatives in perioperative care were considered crucial. Patients appreciated the direct transfer to the rehabilitation and mainly were able to cope with daily life tasks afterward.

**Conclusion:**

In summary, all patients experienced the ERAS protocol positively, and their healthcare process was well established. Active inclusion and education of patients in their treatment can improve patient empowerment. Two further aspects that deserve major consideration in the healthcare process are the inclusion of relatives and interprofessional cooperation.

## Introduction

Enhanced recovery after surgery (ERAS) is an evidence-based, interprofessional, and integrative care pathway designed to reduce the patient’s surgical stress response, optimize their physiologic function, and achieve early recovery for patients undergoing major surgery [1, 2]. With ERAS, patients spend less time in the hospital and the intensive care units (ICU). Therefore, ERAS has a clear potential to reduce healthcare costs [3, 4]. It aims to counteract well-known weaknesses in health care and introduces an improved standard of care. The goal of ERAS is to enhance the recovery process of patients after surgical interventions, resulting in up to 50% shorter post-operative recovery time [5, 6]. Furthermore, patients regain their independence more quickly after surgery. They can return to daily life sooner than patients treated with conventional care protocols [7].

The multi-professional ERAS team usually comprises professionals from surgery, anesthesia and intensive care medicine, nursing, physiotherapy, and nutritional medicine [8]. Perioperative care is individualized and patient-centered, and the involvement of relatives in the treatment process is crucial [7]. An ERAS coordinator (often an ERAS nurse) is one of the major success factors of well-functioning ERAS programs, acting as a central coordinator for all professions involved in the patient´s care [5]. The ERAS coordinator/ nurse is key to ensuring early hospital discharge in the ERAS program [9]. Although ERAS protocols have been established in various surgical disciplines [3], the concept of ERAS in cardiac surgery is still in its early stages. The ERAS® Cardiac Society published its first guideline recommendations in 2019 [10].

### Research gaps

Focusing on the patients´ perspectives on the ERAS approach in cardiac surgery, we identified the following research gaps and implications for practice. Although ERAS follows an interprofessional approach, only physicians have been involved in developing ERAS guidelines. Kubitz et al. [11] were the first to include physiotherapists in developing an ERAS protocol in cardiac surgery, thus following the “real” interprofessional approach. However, the patients are an essential stakeholder group for the ERAS approach that has not yet been involved in developing ERAS guidelines and protocols [12]. Patient experience and feedback are crucial for the successful implementation of ERAS. This study provides the background for including patient feedback in further implementing ERAS protocols.

Moreover, research is required on patients´ experience with ERAS models in cardiac surgery. However, few qualitative studies from other surgical disciplines have addressed patient perspectives in ERAS models [5, 13–15]. Overall, this paper attempts to address a clear research gap in ERAS-based cardiac surgery.

As ERAS in cardiac surgery is still at an early stage, it is crucial to evaluate the interprofessional care process from a patient-centered perspective. In this regard, the study´s qualitative and explorative approach may allow us to derive recommendations for further improvement and raise subsequent research questions.

We aimed to evaluate the newly established innovative ERAS protocol in minimally invasive heart valve surgery at our institution (i.e., university hospital) from the patient’s perspective. Therefore, semi-structured interviews were applied to answer the research question of how optimized health care in cardiac surgery in the frame of an ERAS protocol should look from the patient’s perspective and to derive recommendations for the successful implementation of future ERAS models.

## Background: ERAS protocol

This paper focuses on patients undergoing elective minimally invasive heart valve surgery (i.e., mitral or aortic valve repair/replacement) at our institution who received perioperative care according to our innovative ERAS protocol. The key elements of ERAS were implemented in the care of patients as published by the DGTHG [7] and the INCREASE protocol paper [16]. It consists of the following components:

### 1) An interprofessional pre-operative patient counseling

The first step in the ERAS protocol is an interprofessional pre-operative patient education and counseling session that takes place at least two weeks before the scheduled surgery. Patients and their relatives meet with all members of the ERAS team, including a cardiac surgeon, an anesthesiologist, a physiotherapist, an ERAS nurse (which is an advanced practice nurse specifically trained in the ERAS model), a psychosomatic specialist (usually a psychologist or other trained health professional who addresses the emotional well-being of patients), and a representative from the rehabilitation management (who organizes post-hospital rehabilitation). Patients receive detailed information about the planned surgery and specific information about the ERAS protocol. During the psychosomatic session, coping strategies are jointly developed. Patients are prepared for the possibility of emotional lows after surgery. There is also a focus on setting expectations for the post-operative period to reinforce positive affirmations, and goals are set for the post-operative time.

Furthermore, there is an emphasis on physiotherapy and optimizing physical activity before the surgery.

Moreover, patients are given a carbohydrate- and protein-rich diet ten days before the surgery to improve their nutritional status. Information, education, and empowerment of patients and their relatives are essential aspects of the entire process [5]. They also receive a patient diary with educational information and exercise sheets to complete.

Patient and family involvement and pre-operative education and counseling are essential for successfully implementing the ERAS concept [10, 17].

### 2) A protocol for intra-operative management and post-operative care

The intraoperative ERAS protocol focuses on modified anesthesia and specific surgical details to enable patients to be extubated while still in the operating room. From there, patients are transferred to an intermediate care unit (IMC). Early de-escalation (i.e., early drain and catheter removal) is crucial to successful implementation of the post-operative ERAS.

Moreover, an intensive physiotherapy and mobilization program is essential. Patients receive their first physiotherapy session three hours after surgery and the second six hours later. After one night in IMC, they are transferred early to the regular ward. On the first post-operative day, they receive four physiotherapy sessions. The daily intensity of physiotherapist-guided exercise is then reduced to two sessions. During the hospitalization, the ERAS nurse (together with a psychosomatic specialist, if indicated) takes care of patients’ well-being daily and tries to ensure a well-coordinated, smooth post-operative care process. The frequency of interactions with the psychosomatic specialist was determined based on individual patient needs and clinical judgment, but there was at least one visit after surgery. Furthermore, interprofessional visits are part of post-operative care where the entire ERAS team discusses the next treatment steps with the patient.

### 3) Early hospital discharge directly to the rehabilitation facility

Generally, patients receiving the innovative ERAS model are discharged five to six days after surgery and transferred directly to a rehabilitation clinic. After three to four weeks of rehabilitation, patients should be able to manage their daily lives independently.

## Methods

### Study design & data collection

We conducted semi-structured interviews with patients who underwent minimally invasive heart valve surgery according to the innovative ERAS protocol at our institution. The focus of the interview and questions were in the following categories:

1. Communication and information flow throughout the treatment process
2. Patient care
3. Rehabilitation

Inclusion criteria for the study were: 1) minimally invasive heart valve surgery according to the innovative ERAS protocol at our institution, 2) understanding of the German language, and 3) written informed consent.

We used purposive sampling to select study participants, taking into account the greatest possible variation. We aimed for heterogeneity in the disease course, age, and gender.

Patients were interviewed once. The time of the interview was approximately two to three months after their surgery. Two researchers, trained in qualitative research methods and interview techniques, conducted the interviews by telephone and audio-recorded them using a dictaphone. Furthermore, the researchers maintained neutrality throughout the interview process to refrain from expressing personal biases or opinions and to ensure an impartial and unbiased environment for participants to share their experiences openly. The two researchers conducted test interviews under the supervision of the first author. Patients had no prior interaction or relationship with the researchers before being contacted for the study.

The intention was to interview 10 to 15 patients. From a methodological point of view, as many patients as possible should be included until saturation is reached. Based on this consideration, the recruitment continued until no new findings were obtained from the interviews.

Eligible patients were identified through our cardiac outpatient clinic. We recruited all patients at their scheduled follow-up visits two to three months post-surgery. All participants provided written informed consent before enrolment.

We did not perform repeat interviews or return the transcripts to the participants for comment and/or correction. The interviews were conducted from 01. August 2022 to 01. August 2023.

### Ethics

This study was approved by the ethical committee of the Medical Faculty of the Ludwig-Maximilians-Universtität München on 07/13/2022 (Project Number: 22–0464). Participants gave written informed consent.

### Analysis

The interviews were transcribed verbatim. Data analysis was carried out according to Kuckartz´s structuring qualitative content analysis method [18] using MAXQDA 2022. This method allowed us to employ deductive and inductive coding, allowing for a dynamic approach and adaptations to the research question and categories [18]. Deductive coding involved predefined categories structured around the main elements of the ERAS protocol. Simultaneously, inductive coding allowed new themes to emerge from the data, providing flexibility and openness to participants’ unique perspectives. The analysis is considered mainly manifest as it consists mainly of explicit statements, responses, and observations gathered directly from the interviews.

According to Kuckartz, in the content analysis, the first step involves initiating textual work and marking specific text passages and memos. Following this, main categories are developed thematically, and all material is coded accordingly. The coded text passages are then collected within their respective main categories. Subsequently, subcategories are inductively determined to provide a more detailed understanding of the text. The entire material undergoes a second round of coding, including main categories and subcategories.

The first author, who was not part of the data collection team, conducted the analysis of transcripts, debriefings, and reflexive and observational notes.

### Trustworthiness

In the present study, Lincoln and Guba´s four criteria (credibility, confirmability, transferability, and reflexivity) [19] were used to increase the trustworthiness of the data. The following strategies were employed.

The credibility of the research data was ensured by the researchers´ continuous involvement with the subject of the research and by spending enough time to collect the data. The content analysis method was employed under the careful supervision of the research team according to the study question. All interviews were conducted by trained interviewers.

The transferability of the findings was obtained through a detailed description of the context, participants, environment, and conditions. We tried to select participants with maximum variation as a strategy to enhance transferability. Therefore, we sought to include patients with different experiences during their hospital stay.

Dependability was maintained through the use of a code-recode method during the analysis. While the first author conducted the analysis, the interviewers played a crucial role beyond the interview phase. The interviewers´ prolonged engagement with the data included building rapport, maintaining consistency in the interview process, and recording comprehensive data. Additionally, the interviewing researchers actively built trust and connected with the participants while considering a variety of perspectives. These efforts helped to ensure that the research process remained stable and free of personal bias, which ultimately contributed to the reliability of the research.

To ensure confirmability, the lead researcher who conducted the analysis set aside her previous thoughts and assumptions. Before examining the data, the lead researcher thought about any ideas she might already have about the research topic. She intentionally set those ideas aside and tried to approach the information with an unbiased perspective. Instead of clinging to her initial thoughts, she allowed the patterns in the data to guide her analysis. At the end of the study, peer-check methods were used. Therefore, the transcripts, codes, and categories were sent to two experts in nursing and social sciences to verify the credibility of the extracted categories and subcategories.

## Results

### Sample description

The interviews took place from August 2022 to August 2023. The study team approached 15 patients, and 12 of whom were willing to participate (male = 9, female = 3). Three patients declined to participate in the interview due to time constraints or scheduling conflicts. The mean age was 53 ± 11 years (range = 35–75 years). Five patients had undergone minimally invasive mitral valve repair; three had a minimally invasive aortic valve repair, two had a minimally invasive aortic valve replacement, one aortic valve replacement plus ascending aortic replacement, and one valve-sparing aortic root replacement (David operation). One patient could not be transferred to the IMC after surgery for medical reasons and remained in the intensive care unit (ICU) for one night. The baseline characteristics of our study cohort are presented in Table 1. Table 1 also shows adverse events during the post-operative stay. Mostly, these are minor events that represent typical complications after cardiac surgery and are not associated with the ERAS protocol. Nonetheless, these events are distressing to patients and, therefore, should be considered.

**Table 1:** Baseline characteristics of the study cohort.

Patient	Age	Gender	Surgery	IMC	Considerable events during post-operative stay
1	56	m	DAVID	yes	n/a
2	75	m	MV^1^ repair	yes	n/a
3	35	m	AV^2^ repair	yes	Second surgery
4	60	m	MV repair	no	Atrial flutter
5	39	m	AV repair	yes	Pacemaker Implantation
6	46	w	MV repair	yes	n/a
7	60	m	MV repair	yes	n/a
8	38	w	AV repair	yes	n/a
9	57	m	AV replacement	yes	Pacemaker implantation, retransfer from rehabilitation to hospital due to pericardial effusion
10	62	w	AV replacement	yes	Atrial fibrillation
11	52	m	AV + AA^3^ replacement	yes	Atrial fibrillation
12	54	m	MV repair	yes	Pneumonia, atrial flutter

^1^ Mitral valve

^2^ Aortic valve

^3^ Ascending aorta

### Collected data

**Fig 1:**
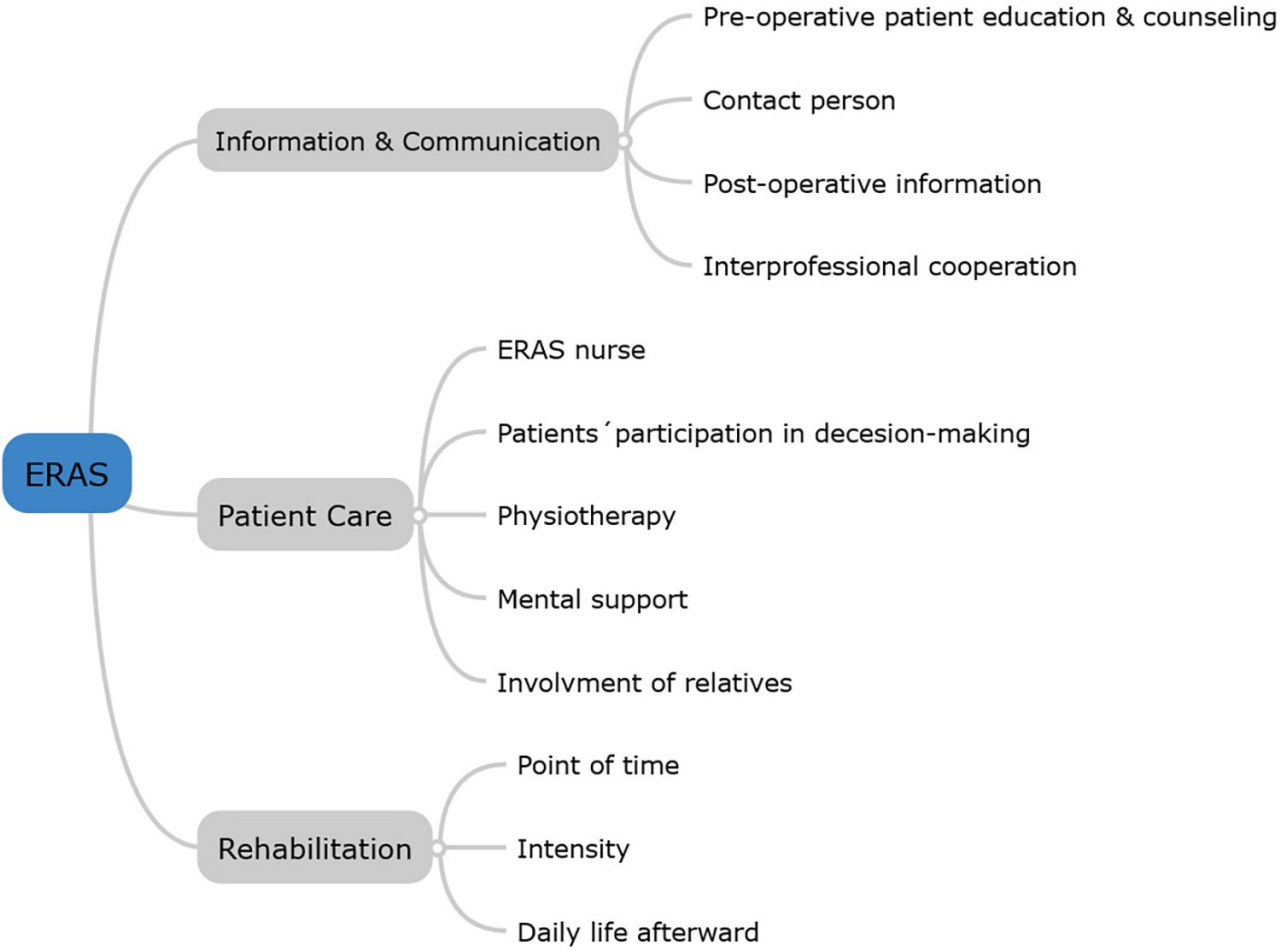
Main categories of the interview with subcategories.

We deductively extracted the following three main categories based on the main components of the ERAS protocol: (1) Communication and information flow, (2) Patient care, and (3) Rehabilitation. These categories are highlighted in the Fig 1. The main statements of the participants presented in this chapter are supported by original quotes from the interviews. The interviews have been translated from German into English.

#### 1) Communication and information flow throughout the treatment process

This main category was divided into the following four inductively built subcategories: (a) Pre-operative patient education and counseling, (b) contact person during the treatment process, (c) post-operative information and communication flow, and (d) interprofessional cooperation.

*a) Pre-operative patients’ education and counseling*. All 12 patients experienced their pre-operative education and counseling day as consistently positive. The timing of pre-operative counseling (two to six weeks before the surgery) was reported to be well chosen. Patients appreciated that there was still time after this appointment to prepare themselves (mentally and physically) for the surgery and to process the information they received during the counseling sessions. Moreover, the content of the counseling sufficiently covered all aspects that were important to the patients (e.g., preparation for the surgery in terms of diet, physical activity, and mental health).

*“(…) I have to say that the whole thing made sense. I could calmly accept the idea that I need heart surgery at such a young age.”*

*"Especially helpful for me was the surgery preparation: factually, what can I do to optimize the surgery process—with food, with movements, with exercises? I found that helpful."*

The positive aspects of this counseling day were, for example, the perception that there was enough time planned for each profession, which, in turn, gave the patients a feeling of security. Furthermore, the fact that the patients met all the professionals involved in the treatment process and the faces of these people were found to be relieving and reassuring. The structured process and "knowing exactly what to expect" appeared to have the same effect.

*"Yes, I also thought it was good that there were different professional groups there. I got to know the people a little bit, and I got many explanations in advance about what would happen and how it would work. (…). That’s something that makes me feel relatively well taken care of (…). This predefined structure, knowing exactly what to expect, was helpful."*

*"In the beginning, I had real psychological problems with this surgery. Yes, but it really was better after the explanatory talk. (…) And that I saw familiar faces again before and after the operation. That reassured me a lot."*

Moreover, patients reported that psychosomatic counseling and mental preparation helped to reduce their anxiety.

*"The most useful thing for me was the discussion with psychosomatics, i.e., how the mental course could be. That there can be lows after the surgery. But this is also quite normal, and it usually gets better continuously. And also to see the whole thing in a positive light."*

*“I was terrified of the surgery. But this preparation helped me well. The psychological stuff, too. And then, I could contact the psychosomatics specialist again before the surgery. That was also good because sometimes I was petrified. That calmed me down again. In any case, the preliminary education also reduced my fear of the operation.”*

Less information during the pre-operative visit would have been acceptable for some patients. Some patients experienced the education and counseling day (which usually lasted 5 hours) to be quite exhausting but still important.

*"(…) I often thought afterward, "Boah, I didn’t want to know exactly where the tubes come out and what can happen. (…) It [the pre-operative education] was very exhausting.“*

*b) Contact person*. The ERAS nurse was the primary contact person for all ERAS patients. All interviewed patients were aware of this and made use of it.

*“So mainly I got information from the ERAS nurse during her daily visits… that’s where most of the information came back. There was also information from the ward staff and partly from the doctors. But most of all, I would say, really about the ERAS nurse.”*

*"And the ERAS nurse was always there with advice and support, and I was always well informed, yes. Because what I always found a bit strange about other hospital stays is that you often don’t have any information status—about what happens next or something like that—and that was just great."*

The ERAS nurse was the person of trust for all the patients and the primary contact person for any issues and questions. Patients felt very comfortable with the ERAS nurse and dared to bring up things or ask questions they would have been afraid to ask a doctor. Furthermore, the ERAS nurse provided all relevant treatment information to the patients and played a central role in communication.

*“That [the ERAS nurse] was extremely helpful. Because I could always ask questions if something wasn’t clear. I always got an answer. And you weren’t alone but always had someone to explain things to you. That you knew who you could come back to and to have a fixed contact person.”*

*c) Post-operative information*. Even though the ERAS nurse was usually available as a contact person, some patients would have appreciated more information about the course of the surgery directly from the surgeon.

*“Where I struggle a bit (…) is that after the surgery, there came never anyone who had been there during the surgery. So, there were always just residents. And I would have had a few more questions for the surgeon.”*

*“The only suggestion for improvement would perhaps be that the surgeon (…) had come to the ward round again (after the surgery) and told me: "Everything’s great."—I would have found that super, yes.”*

Especially on weekends, when the ERAS nurse was not available, some patients reported a need for more information. In particular, when unforeseen complications or events occurred (e.g., pacemaker implantation or retransfer from rehabilitation to hospital).

*“So what set me back was that I also needed this pacemaker. I was operated on a Wednesday. And then, they found out on Friday that I still needed the pacemaker. I was waiting all Friday and Saturday for it. Somehow, it was impossible to install it, and then I did not feel well, and there was very little communication with me. (…) So this waiting time and the uncertainty really didn’t do me any good."*

*d) Interprofessional cooperation*. The entire ERAS process is based on intensive interprofessional cooperation. Patients met all the involved professions at the pre-operative counseling and remained in contact with them during their hospital stay. Patients’ perceptions of interprofessional cooperation were heterogeneous. Some patients were positive about the interaction among all the professions involved.

*“It seemed to me that they work very hand in hand. As I said, nursing staff and then physiotherapists with ERAS ladies [ERAS nurse, psychosomatic specialist]. So everything was actually already coordinated, and I had the feeling that that goes hand in hand."*

On the other hand, some study participants were not consciously aware of this or even experienced insufficient communication pathways among the different professions. Interprofessional cooperation was not as important for patients for whom everything went according to plan and went well. It seemed to become more relevant when things were not going well.

*“Well, I have to say that I didn’t really perceive that [the cooperation among the professional groups]. But everything went well, so it worked.”*

*"Sometimes I certainly would have liked a little more communication, probably between doctors and ward staff."*

#### 2) Rehabilitation

This main category was divided into three subcategories: a) point of time, b) intensity, and c) daily life afterward.

*a) Point of time*. As part of the ERAS program, patients are transferred directly from the hospital to the rehabilitation facility. Most patients found this tight schedule to be supportive in their recovery process. Furthermore, patients reported feeling safer in the rehabilitation center than at home without close medical surveillance. On the other hand, patients were away from home for up to four or five weeks, which they felt was a long time.

*"For me, it was so good to continue right away. If I had been at home for a few days or maybe longer, I wouldn’t have known what to do. That would have been a break. (..) And so it went right on in the process that had already started in the hospital. (…) Of course, it’s also a long time since you’re not home."*

*"Well, I found that very pleasant. (…) So maybe one or two nights at home—I wouldn’t have considered that as bad, but to get directly and quickly into rehab, that’s very important, in my opinion. (…) Because if you first get out of the hospital after such a surgery (…). That would have scratched my psyche quite a bit (…).”*

Additionally, patients described the direct transfer to rehabilitation as a relief for their relatives, who would have been overwhelmed if they had to care for them at home.

*"So that was really great for me and, first and foremost, for my husband and the other relatives. It was ideal because–(…) in the first few days in rehab, there were often situations (…) where you suddenly have heart palpitations or a situation where you think, "Wow, is that normal now?". So, if I had been at home, I would have been worse able to deal with it. And in fact, so would my husband, because in that case, he doesn’t know what to do either. Above all, he’s not available the whole day (..). I think it was even better for him because he had a calm feeling that I was in good hands. (…) I would not have been able to cope so well with some situations at home, probably without calling either a doctor or an ambulance (…). So the uncertainty is completely taken away from you.”*

One of the interviewed patients felt that his discharge was too early and he didn’t feel ready for rehabilitation. It is worth mentioning here that this patient had pneumonia after surgery and did not feel as well as other patients at the point of discharge.

*“I was discharged from the hospital too early, I guess. Or did I agree too early? (laughs)”*

One of the interviewed patients had to return to the hospital for one night from rehabilitation. Nonetheless, he felt positive about this early discharge and wanted to leave the hospital as soon as possible. Due to the medical care provided at the rehabilitation clinic, his complications were quickly identified, and a rapid reaction was possible.

*“On the one hand, I was happy to get out of the hospital as soon as possible. (…) But it was very bumpy initially. (…) I came to rehab on a Wednesday, and on Thursday (…) in the evening, I already had a fever, so those were the first signs that something wasn’t quite right.”*

*b) Intensity*. Most patients were treated at the rehabilitation clinic for three or four weeks, which is the same length of time that non-ERAS patients would stay there. Most patients found this to be a reasonable length of time. In most cases, patients continued to get fitter during this time. Notably, the rehabilitation clinics did not implement a specific ERAS protocol, although they tried to tailor their programs to the patient´s individual needs.

*“The rehab lasted a total of four weeks, yes. And then you just noticed how it was going uphill bit by bit, and I felt that was exactly what I had expected—and that was pleasant. But at some point, after four weeks of rehab, you slowly want to go home again.”*

For most patients, the intensity of the rehabilitation program was just right.

*“So I was able to do everything at the rehab from the beginning without any problems. So well, the preparation for the surgery was very good.”*

*"The rehab was positive throughout. (…) At that moment [where I arrived at rehab], it was the case that I thought to myself, "Oh my God, this is all very much." And I couldn’t do anything—but after two to three days (…) I saw that compared to other patients, yes, I’m actually relatively far along with the recovery process already."*

One of the interviewed patients found it hard to participate in the therapeutic program as he still had problems with his chest discomfort.

*“But I couldn’t do much [except for cycling at the ergometer] then because I couldn’t put any weight on my sternum. Yes, so everything was a bit more difficult. The medical care at the rehab was great.”*

*c) Daily life afterward*. Most patients reported being able to function independently in their daily lives after rehabilitation.

*“I felt (…) that I was in good shape after rehab, yes. I’m still nowhere near one hundred percent, but I’m already doing well, yes. So, as I said, I am fit enough that you can cope with everyday life and that I can do everything independently at home again. So the rehab has put me back on my feet."*

*“And after a few weeks of rehab, I almost felt healthy again.”*

Only a single interviewed patient reported that he did not feel fit after rehabilitation due to complications. This patient also had to return to the hospital for one night during his rehabilitation stay.

*"I wasn’t fit after the rehab. I noticed that relatively quickly, on the day of discharge, or the day after—colloquially, I would have said, it pulled the plug on me in the afternoon."*

#### 3) Patient care

This main category was divided into five inductively created subcategories: (a) ERAS nurse, (b) patients’ inclusion in treatment, (c) physiotherapy, (d) mental support/ psychosomatic, and (e) involvement of relatives.

In general, ERAS patients were satisfied with their healthcare process in the hospital. Despite the negative perception of hospitalization in general, patients in the ERAS program reported a positive experience during their stay. They rated the care they received as health-promoting.

Patients were able to contact the ERAS nurse and the psychosomatic specialist after their hospital discharge. Although no specific time limit was set, most patients contacted them within the first three months after hospital discharge. Later contact was rare.

*a) ERAS nurse*. As mentioned above, the ERAS nurse was the primary contact person and played a central role in the care process. Patients stated that the ERAS nurse had more time for them than the regular nursing staff on the ward. For example, they had enough time to help patients take a shower.

*"I can still remember when I simply said, "Well, the nicest thing for me right now would be to just get in a shower again after a few days." And then the ERAS nurse said, "Well, that’s not an issue, we’ll help you with that." Because the normal staff couldn’t do it, I alone couldn’t do it either, yes, and in this respect, it was simply a dream, yes."*

Furthermore, patients appreciated the ERAS nurse as a consistent person of trust, in contrast to the daily changing ward personnel due to different shifts.

*“There was a familiar face there immediately after the surgery. I was really well taken care of.”*

*“And you didn’t stand there alone but always had someone to explain things to you. You knew whom you could return to and had a fixed contact person.”*

Moreover, the patients reported feeling well cared for and being noticed” as a human being. The presence of the ERAS nurse also made patients feel less alone in the hospital. The interview responses indicate that the ERAS nurse played an important role for the patients, particularly on an interpersonal level.

*“My ERAS nurse, in particular, gave me a lot of information. She always took good care of me. She took a lot of time for me. And she was always with me. I could always turn to her. But also the doctors and the professor were with me. I felt well cared for. (…) Everything was organized for me. And that was great”.*

*“I didn’t just feel like a number but as a patient being treated and cared for. And that is something that was very important and good for me. I had the feeling that I was being noticed. Not just as another patient, but as a person.”*

*b) Patients’ participation in the decision-making about treatment*. Most of the interviewed ERAS patients reported positively about their active involvement in their treatment and the decision-making process. The key factor was that the ERAS nurse (and other professionals involved) took enough time to explain the following steps to the patient.

*“So I was also able to ask questions, so I never felt like I was somehow, yes, practically not noticed.”*

*"I couldn’t have wished for it any better. You’re not just a stranger in the hospital, but you’re a patient. And it’s clear to the others what’s happening to you (…). And that this is also something big for the patient. That made me very happy, and I wish it would always be like that in the hospital and rehab."*

*c) Physiotherapy*. Most patients perceived fast mobilization after the surgery as beneficial and encouraging.

*“I have to say, quite honestly, that the physiotherapists are also very helpful. It is advantageous when accompanied by the physiotherapists from the second [or third] hour after the surgery. Where you might be unsure yourself—"What am I allowed to do now?" or "Am I allowed to walk, stand up, whatever?" They have always encouraged me to do that, and then I also dared to do it.”*

Furthermore, the physiotherapy encouraged patients to move and get out of bed. This was a motivating factor for the patients to exercises to get fit quickly and support the healing process. Even though ERAS patients received more physiotherapy than conventional care, it was not rated as too intensive.

*“The physio was a little more intense than the normal stuff—that helped me to get on my feet a little more, of course, than if I hadn’t had that.”*

*“It’s just that it was also quite a good motivation and support to overcome this sluggishness and get going again.”*

*d) Mental support*. As the primary contact person, the ERAS nurse helped patients to feel well cared for and safe. Furthermore, patients have generally felt well supported by the care provided by the ERAS nurse and the psychosomatic therapist. Even though minimally invasive, heart valve surgery was a mental challenge for patients. Because of this, all patients described the psychosomatic preparation and support during the process as helpful. Some patients used it more than others. The frequency ranged from several visits a day to only one after surgery. Those patients who were very anxious about the surgery or felt nervous benefited from it even more.

*"A psychologist called me again before the surgery because I was very nervous.”*

*“Yes, both the woman from psychosomatics and the ERAS nurse were there almost every day during my first hospital stay and also when I was transferred back [to the hospital] the second time, um—I don’t know how often they were there—but they (…) looked after me, talked to me, discussed various things and gave me courage. So that was (…) very good. Especially when you might have had a low point or something, where they helped you overcome it, gave you courage, and straightened you up.”*

The coping strategies that were addressed during the psychosomatic education were perceived as valuable. Moreover, the detailed information and the knowledge that a mental low is normal after surgery was very helpful.

*“That was also great because (…) I am a bit unstable in psychological matters. The [psychosomatic specialist] gave me a lot of information beforehand and also practically took care that I was psychologically cared for in the rehab. Therefore, that was a bit of a red thread running through it.”*

*“And, what I also found good is [that] we have made a list [patient with psychosomatics]. What could occur, like pain, fear, whatever. And then, we always tried to find possible solutions or behaviors that could be used to counteract this. That helped me most of the time. (…) she [psychosomatics] had named some things, where I would not have thought about at that moment, that this could happen. And in this respect, that was very good.”*

*e) Involvement of relatives*. The ERAS patients were allowed to bring a relative to the pre-operative counseling day. All patients who took advantage of this opportunity experienced it as beneficial. Patients perceived their relatives as an additional backup who was just as likely to listen, ask questions, and remember information.

*“It was really great that my husband was also able to be there [at the pre-operative education]. In such moments, you’re often a bit nervous yourself or forget some of the questions. And if your partner is there, he can actually stand in for you or have other questions. So I found that very pleasant.”*

Furthermore, it was also valuable for the relatives themselves. In some cases, patients reported that their relatives were even more nervous than the patients.

*“Yes, well, my husband has taken it relatively relaxed. Or rather, he is simply pragmatic. But my mother was much more nervous than I was before the surgery.”*

In such cases where relatives were distraught, their participation in pre-operative counseling calmed them down and made them feel better than before.

*“After my wife has been present at the [pre-operative] conversation, she was also actually reassured.”*

*“For my wife, it was simply important to get the information first-hand. And when she has a question, that it will also be answered. (…) Well, it helped her in any case.”*

The relatives were also encouraged to contact the ERAS nurse or the psychosomatic specialist during the care process or afterward if they had any questions or concerns. This contact possibility and the involvement of the relatives were highly appreciated. Furthermore, this support was also a mental relief and help for the relatives.

*“Yes, my wife was even present at the information meeting. (…) My wife also sought contact with the woman from psychosomatics, and that worked wonderfully, so in the end, she was neatly integrated. And otherwise, of course, my family also received information from me that I had received before, yes—but they were involved.”*

*“Well, especially my sister, with whom I do a lot together, she was quite well involved, yes. And she was also, uh, always informed accordingly and, uh, she also had to struggle quite a bit with the situation. And she was also quite well looked after. Yes, so that was actually a great thing.”*

## Discussion

This study aimed to evaluate the implementation of the newly established innovative ERAS protocol from the patients’ perspective. Furthermore, we aimed to examine the patients´ experiences and expectations of a newly developed ERAS program in cardiac surgery.

Overall, our findings indicate that ERAS patients were mainly satisfied with their perioperative health care. The most relevant aspects for optimized health care in cardiac surgery in the context of ERAS are the pre-operative education and counseling day, the holistic approach (incl. ERAS nurse, physiotherapy, psychosomatics, involvement of relatives), a well-functioning information flow (having a primary contact person, receiving all relevant information) and prompt rehabilitation.

These main topics are summarized and discussed in more detail in the following paragraphs.

Our results show that the ERAS program in our hospital incorporates patient-centered elements in several key aspects by prioritizing individualized information, involving patients in decision-making, promoting interprofessional cooperation, and recognizing the importance of social support from relatives [20]. The review by Kitson et al. [21] identified three main core elements that are critical to patient-centered care: 1) patient participation and involvement, 2) the relationship between patient and health professional, and 3) the context in which care is delivered [22].The pre-operative education and counseling day is the first crucial step in successful patient-centered perioperative care. Our qualitative study highlighted three major components that are of utmost importance to patients. First, detailed and holistic perioperative information is essential. Medical information should be comprehensive and patient-centered [20]. In order to ensure patient-centered perioperative care in ERAS, it is crucial to customize the information provided during the pre-operative education and counseling day and focus on the individual’s specific healthcare needs [23]. For example, we have found that not every patient wants to receive such detailed medical information. Therefore, it may be helpful to clarify the appropriate amount of information for each patient in advance.

Furthermore, information on nutrition, exercise, and mental health information greatly enhances patient preconditioning. This, in turn, leads to the second important aspect of the pre-operative education day: by providing this holistic information, patients are given the opportunity to prepare themselves and actively contribute to the healing process after surgery. This can lead to increased self-confidence and patient empowerment. Patient empowerment is a core value of high-quality patient-centered care [24, 25].

Third, the pre-operative education day, including mental preparation, had a calming effect on the patients. The distributed coping strategies for unpleasant situations may give the patients the self-confidence that they have things under their control. However, this hypothesis lacks direct support from the available literature and should be evaluated in future research [26].

Heart valve surgery, even though minimally invasive, represents a mental burden for the patients and their relatives [27], which our findings confirmed. Therefore, psychosomatic counseling and mental preparation during the pre-operative patients’ education are crucial, and we recommend including them in any ERAS protocol. Salzmann et al. [28] have already shown in their PSY-HEART trial that pre-operative optimization of patients’ expectations can significantly improve the outcome of cardiac surgery. Moreover, mental and psychosomatic support is crucial throughout the healthcare process. In general, we would recommend the implementation of psychosomatic or psychological support for all patients undergoing cardiac surgery, such as coronary artery bypass graft (CABG) surgery, as the review by Robinson et al. [29] shows that cognitive decline after CABG surgery is common. Moreover, there is clear evidence that cardiac surgery can lead to postsurgical emotional difficulties [30–32]. However, reviews are confirming that psychological treatments and pre-operative education have a positive impact on anxiety and depression levels for patients undergoing cardiac surgery [33, 34].

A constant and up-to-date flow of information was a crucial element for patients to perceive their health care as optimal. The fact that they had the ERAS nurse as their primary contact person resulted in a positive assessment of the communication and information flow throughout the treatment process. Patients were more likely to miss appropriate information when the ERAS nurse was away from the clinic, such as on weekends. Therefore, having a primary contact person is a decisive factor for optimal care. Especially in ERAS programs, having a well-coordinated treatment plan and functioning communication between all professions is elementary. Nonetheless, many other interprofessional healthcare processes would likely benefit from a central person coordinating all processes. Our results are consistent with the literature, stating that the ERAS nurse is more than just a coordinator [35, 36].

One area for improvement in the communication flow was for patients to receive post-operative feedback from their surgeon. In our hospital, this usually happens in the first 24 hours after surgery, when patients are still in the IMC. Patients may not be able to remember details of what happened in the immediate post-operative period. Therefore, a practical solution could be the implementation of short surgical feedback by the operating surgeon immediately before discharge from the hospital. There is a lack of literature on post-operative feedback to patients by their surgeons.

Our findings regarding high-intensity physiotherapy, starting three hours after surgery lead to a general recommendation for more post-operative physiotherapy in the cardiac unit. A possible solution to increase the intensity of exercise could be group physiotherapy. The positive feedback on physiotherapy also implies that it is essential to patients’ empowerment and patient-centered care, which is in line with existing literature [37, 38].

Moreover, the supervision by physiotherapists provides great advantages for the healing process through the encouragement of exercise and confidence in what the body can afford after surgery. This enables a much faster healing process for insecure patients who tend to fall into protective behaviors. The review by Abeles et al. [39] showed that the amount of physical activity performed after surgery was inversely correlated with the length of hospital stay.

There is a need for support from family members during the perioperative process, as demonstrated by several studies [40–43]. Moreover, the involvement of relatives in the education and treatment process is beneficial for the relatives themselves, as the surgery is associated with psychological distress due to anxiety about their loved ones [40]. This can add to patients´ stress if they have to worry about their relatives on top of their anxiety which raises the consideration of involving relatives even more in health care [41]. One way to achieve this could be to provide a constant point of contact for relatives. Furthermore, relatives could be more involved in pre-operative education and post-operative interprofessional visits in the clinical setting. Moreover, digital solutions may be possible as well. For example, Pozzoli et al. [44] implemented an electronic communication solution to establish daily communication with patients´ families during the COVID-19 pandemic.

We recommend implementing daily interprofessional visits because they can have several benefits. Firstly, they enhance patient involvement in their treatment decisions [45]. Secondly, they can promote and represent interprofessional cooperation, further strengthening communication among the healthcare team [46, 47]. Additionally, involving relatives in these daily visits could provide a more comprehensive understanding of the patient’s journey [45].

Another implication for the future could be to facilitate communication between the hospital and the rehabilitation facility. Specifically, an exchange between physiotherapists and psychologists/psychosomatic specialists in both settings could be advantageous regarding continuous and trans-sectoral health care that promotes a holistic and seamless experience for patients. Future interventions and protocols should, therefore, not only prioritize effective communication channels but also emphasize the seamless transfer of care responsibilities to provide patients with a cohesive and personalized healthcare experience. These transitions involve not only the transfer of medical information but also the coordination of interprofessional efforts to ensure a smooth progression of care. Facilitating successful care transitions is a key nursing competency [48]. In addition, a coordinated team approach to care transitions is essential [49]. The interprofessional ERAS team, with the ERAS nurse as the coordinator, already has the essential components for successful care transitions. However, there is considerable untapped potential in this area.

Patients supported the direct transfer from the hospital to the rehabilitation facility and were aware of its benefits. This is the main difference between ERAS and standard care in terms of rehabilitation. With standard care, patients can experience several weeks of waiting time, which allows ERAS patients to return to daily life and work more quickly than with standard care.

Some patients who were away from home for up to five weeks preferred to go home for a night or two before going to rehabilitation. Going home has pros and cons: Going home to familiar surroundings with close relatives may benefit patients’ mental well-being [43]. Nevertheless, this is precisely the time when patients report feeling insecure about what is allowed and what is harmful to the healing process. However, going home is only advisable if relatives provide adequate support and patients and relatives feel safe enough to handle any situation at home [50].

### Limitations

We are aware of several limitations of our study design. Firstly, data were obtained from only one hospital. Patients’ experiences may differ in other hospitals because different ERAS protocols exist and vary from hospital to hospital. Nonetheless, the insights from our qualitative study may guide other hospitals seeking to implement an ERAS protocol in cardiac surgery.

Secondly, it is important to note that the generalizability of the findings may be limited due to the nature of qualitative research, which focuses on in-depth exploration rather than broad representation. Future research should include a broader range of perspectives to achieve a more comprehensive and applicable understanding of diverse patient populations. For example, including patient-reported outcome measures may achieve a more nuanced and representative view of patients’ experiences with ERAS protocols in cardiac surgery.

Thirdly, in our clinical setting, heart valve surgery is more commonly performed on male patients than on female patients, making it challenging to achieve a balanced gender representation in our study sample. Despite efforts to accurately reflect the gender distribution in our patient population, the available pool of eligible participants primarily consisted of male patients.

Certainly, when working with patient feedback, it is crucial to be aware of potential biases that may influence the interpretation of results. One notable aspect is the use of purposive sampling in our study, which may introduce selection bias. The experiences of those who volunteered to participate may differ from those who chose not to participate. Moreover, the focus on patients undergoing minimally invasive heart valve surgery within the context of an ERAS protocol introduces biases associated with the specific surgical procedure and protocol. It is important to recognize these biases to assess our results’ applicability and generalizability accurately. While our study is susceptible to recall bias, given the challenge of accurately recalling specific details, particularly past events, this potential limitation is inherent in studies that rely on retrospective reports. Therefore, the interviews were strategically timed at 2–3 months post-surgery for practical recruitment reasons. This post-surgery window, occurring shortly after rehabilitation completion, aimed to balance practical considerations with the desire for participants to provide rich and reflective insights.

### Future research

Future research should investigate the long-term outcomes of patients undergoing cardiac surgery with an ERAS approach. Studies should include assessing not only immediate post-operative outcomes but also the ongoing impact on patients’ quality of life, functional capacity, and overall well-being over an extended period of time.

Valuable insights could be gained by exploring diverse methods of delivering perioperative information to meet individual patient preferences. Research could involve evaluating the effectiveness of multimedia resources, mobile applications, or personalized counseling sessions to determine the most effective and patient-centered approaches.

Another area of research is the involvement of relatives in the perioperative process. Understanding how involving and educating family members contributes to patient outcomes and adherence to ERAS protocols could enhance the overall support system for patients undergoing heart valve surgery.

In addition, including a complete set of Patient-reported Outcome Measures (PROMs) to capture various patient-reported outcomes, such as physical, emotional, and social aspects, would provide a more comprehensive understanding of the impact of heart valve surgery and the related perioperative care on patients’ lives.

In future studies on ERAS, it may be of great value to evaluate patient empowerment to measure the program’s impact in promoting patient autonomy and active participation. This evaluation will provide useful insights for refining protocols, building patient trust, and tailoring interventions to improve perioperative care.

## Conclusion

In summary, pre-operative intensive and holistic education provides the foundation for optimal ERAS care. In addition, it may enable patient empowerment. Constant and clear communication and information flow are essential throughout the treatment process. A primary contact person, such as an ERAS nurse, is accommodating to ensure this. Moreover, the interprofessional care provided by nurses, doctors, psychosomatic specialists, and physiotherapists is essential for optimal ERAS-based protocol.

Implementing the innovative ERAS protocol in our hospital can be considered successful from the patients’ perspective. Further improvements would make health care after cardiac surgery optimal from the patients’ perspective.

## Supporting information

S1 File Anonymized code segments of interviews. (PDF)

S1 Fig (TIF)
