# Supplementary material for: Implementation of an innovative ERAS protocol in cardiac surgery: A qualitative evaluation from patients’ perspective
Source: PLoS One. 2024 May 10;19(5):e0303399. doi: 10.1371/journal.pone.0303399 (PMC11086837; doi:10.1371/journal.pone.0303399)
Supplement: S1 File — (PDF) [file pone.0303399.s001.pdf]

Supporting Informtion_S1 File_ Anonymized code segments of interviews

Sorted by themes.
Patient IDs are deleted in order to secure anonymity. Original language was German. Transcripts were translated into English. The codes are randomly ordered by themes, as a provision of full interviews would identify patients.

I (Interviewer)

How did you experience your overall stay in the hospital?

B (Interviewee)

(Laughs) Well, how should I put it, um, like a hospital stay is supposed to be. So, that was - I felt well taken care of and, um, I didn't lack anything. The surgery also, let's say, went well - I had a little heart flutter afterwards, which was a bit critical, and I was afraid, I must say. So that was very stressful. And, um, yeah, and then of course, for me, the biggest limiting factor was the pneumonia I had after the surgery. (I: Yes.) It was diagnosed five days after the surgery - or four. And I was practically discharged to rehab - with pneumonia - and when I got there, I could hardly walk, yes. So that was - they wanted us to do sports there and I could hardly get up - that was a bit difficult. If you ask me, I was discharged from the hospital too early, I guess. Or did I agree too early? (laughs)

Code: ● Patient Care

IG Position: 71 - 73

I

Were you overall satisfied with your treatment and care?

B

Yes.

I

Okay. So, do you feel that this process contributed to your recovery?

B

Absolutely. If I look now, the surgery was about three months ago - just about - and my current state of health is so good that the doctors always ask me in disbelief when my surgery was. So, definitely, um, let's say, faster than apparently usual - my recovery.

Code: ● Patient Care

IG Position: 146 – 153

B

Um, I'll put it this way, within that context, it was fine. In terms of care, overall, it was good care, there on ward [redacted] - I can't complain. If there was anything, the employees were always helpful, um, and so I have to say, that was good. Regarding ERAS, as we've already discussed, that was very good. If I have to criticize something, I would say, then it's the food.

Code: ● Patient Care

IG Position: 62 - 62

B

But well, I mean, that may be a personal thing, um, and there are certainly more important things - so the medical care was good so far, and yeah, I think that's the most important thing.

Code: ● Patient Care
IG Position: 66 - 66

B

During my hospital stay, I had a contact person from ERAS who provided me with more attention and support than the regular ward staff. This was especially helpful during low points when they encouraged and uplifted me. They also assisted with nursing care, and I can still remember when I simply said, "Well, the nicest thing for me right now would be to just get in a shower again after a few days." And then the ERAS nurse said, "Well, that's not an issue, we'll help you with that." Because the normal staff couldn't do it, I alone couldn't do it either, yes, and in this respect, it was simply a dream, yes. There were many other points to consider, but I would like to highlight the ERAS team who provided me with care during my hospital stay. Their service was commendable and top-notch.

Code: ● Patient Care
IG Position: 70 - 70

And now, from the treatment perspective, or rather from the medical perspective, one must say that a big support was indeed the ERAS team - one simply has to say that, yes. Um, especially in the beginning. And in rehab, one must say that there were some therapists who worked there, of course.

Code: ● Patient Care Gewicht: 0
IG Position: 210 - 210

The care [in ERAS], um, was more intensive, also the rehab and the physio - or specifically the physio. Right after the surgery, that was indeed a bit more intense - yes.

Code: ● Patient Care
IG Position: 10 - 10

I felt well taken care of, except for at the beginning of March, when the surgery was postponed by a month. That was a pretty big blunder, so to speak. When the surgery actually happened, I did feel well taken care of again. But in terms of the clinic's organization, it was - you were just a number, where you were more or less processed - beforehand. During the clinic or something like that, it was good again. (I: Okay.) But before that, it was - it wasn't a great move that the surgery was postponed again.

Code: ● Patient Care
IG Position: 70 - 70

And the ERAS nurse was always there with advice and support, and I was always well in-formed, yes. Because what I always found a bit strange about other hospital stays is that you often don't have any information status - about what happens next or something like that - and that was just great.

Code: ● Patient Care

IG Position: 94 - 94

Ultimately, it was also the kind support from ERAS that made a big difference, yes. So I can only express praise for that, yes. And in general, I was also quite well taken care of by the nurses. Yeah, and otherwise, well, yeah, they had their rounds - they came by or something like that. And before, I was properly informed by various doctors about what's going on and what needs to be done - if decisions need to be made during the surgery. I was properly informed beforehand. And afterwards, from the medical point of view - well, they did their rounds and, um, when there were complications, I was properly informed and then things happened quickly, yes. And, well, I can't really say that decisions were made about the surgeries and after the surgeries without consulting me - the situation just arose, and accordingly, reactions had to be made, so yeah. (I: Okay.) So I see it quite pragmatically, yes. One could have also said, there are complications and, well, I don't want to be operated on. But there wasn't a big decision to make, yes.

Code: ● Patient Care

IG Position: 118 - 119

Um, so the ERAS support actually boosted my acceptance of treatment during my stay at the hospital a bit further, yes. So I just found it good for what it is, and um, I definitely found it better than if it were the classic standard treatment - which is surely okay as well, yes. But I did perceive it as advantageous, and in terms of expectations - the opinion is that it went up another notch when experiencing it firsthand.

Code: ● Patient Care

IG Position: 178 - 178

I

All right. Then, personally, do you have things or measures that you would now claim were the best or most positive for your treatment?

B

The support from the ERAS ladies, that was really good and that was my personal favorite.

Code: ● Patient Care

IG Position: 212 - 214

So, I was better supported, informed beforehand about what happens during the surgery. I could really talk to the anaesthetist, the surgeon, with the social worker beforehand. I had psychosomatic support and was never alone afterwards, constantly accompanied, both physically and emotionally. The physiotherapist came just two hours after the surgery, got me up and cycling the next day. So in that sense, I think it was just a more intensive preparation, follow-up, and support.

Code: ● Patient Care

IG Position: 14 - 14

I

How did you experience your overall stay in the hospital?

B

So, very pleasant, I must say. Whether it was the ERAS ladies, the surgeon who came to see me, the doctors - in this case, it was the [redacted] Then the entire nursing team, I really have to say, was accommodating, friendly, and they really addressed every wish or issue I had. So, all around great - really.

Code: ● Patient Care

IG Position: 52 - 54

I

Okay, I would like to go through the different professions a bit. If you remember again, the ERAS nurse who took care of you. Do specific things come to mind, or just, yes, a feeling associated with that?

B

Yes, well, primarily there's just the feeling that I didn't have to go through it all alone. So I was really glad that there was actually someone there.

Code: ● Patient Care

IG Position: 56 - 58

I

Okay, was there a person or perhaps a professional group that particularly helped you there?

B

Yes, specifically the nursing staff - also the physiotherapists, I have to say, quite honestly, that the physiotherapists are also very helpful. It is advantageous when ac-companied by the physiotherapists from the second [or third] hour after the surgery. Where you might be unsure yourself - "What am I allowed to do now?" or "Am I allowed to walk, stand up, whatever?" They have always encouraged me to do that, and then I also dared to do it.

Code: ● Patient Care

IG Position: 76 - 78

Expectations, well. Firstly, I actually thought I would have a longer hospital stay, that overall it might be a bit slower. But actually, through the intensive care, you dare to do more yourself and then also achieve more.

Code: ● Patient Care

IG Position: 126 - 126

So, let's say, the hours after I was transferred from IMC to the regular ward - firstly, there's a gap from the last day in Intensive Care and all that - and I have to say, it was almost a bit too much. Because every half an hour, someone came - the lady from the rehab management, then another lady from ERAS came again in half an hour later for another measurement. And I thought to myself:

"Oh my God, I'd just like to sleep for maybe three hours now." So, those first few hours on the regular ward, I found them exhausting.

Code: ● Patient Care

IG Position: 130 - 130

And yes, what I really appreciated the most is - the support from the ERAS nurse and the psychosomatic specialist.

Code: ● Patient Care

IG Position: 158 - 158

I

Is there anything you would have wished for in this entire process that wasn't there?

B

No. No. Looking back now, I've also met other heart patients during rehab. Or I've also been in contact with two people who were also in Augsburg. And I have to say, it went really well for me.

Code: ● Patient Care

IG Position: 52 - 54

But I didn't think beforehand that the care would be so much better for me.

Code: ● Patient Care Gewicht: 0

IG Position: 95 - 95

So just the intensive care, something like that. Hm, well, I found it pleasant.

Code: ● Patient Care

IG Position: 46 - 46

I

Okay. Um, was there anything during your stay here that didn't go so well?

B

I would say no. So what I found more positive is that after the surgery, you just feel dirty and your hair sticks, you know. And, um, that I could shower and also had help afterwards. Normally, they don't have so much time for that in everyday hospital life.

I

Who helped you then?

B

Yes, that was the ERAS nurse.

Code: ● Patient Care

IG Position: 68 - 70

So, I really enjoyed the relatively short stay. Surgery on Tuesday, discharged on Monday. That was very pleasant, I think, as a patient. And otherwise, as I said, all the procedures, how everything happened. I found all of that very pleasant. It was truly a nice stay, almost like a vacation.

Code: ● Patient Care

IG Position: 135 - 135

I

I would ask another question about the ERAS nurse. How much contact did you have with them?

B

So I would say quite a lot. Right from the beginning, when I arrived there, even before that, over the phone. Then also during all the conversations, she was always there too. So I would definitely say I had a lot of contact. But I always counted on that. So that worked out. (I: Okay, great.) I would say they also get along well with the other patients, right?

Code: ● Patient Care

IG Position: 171 - 173

Everything went smoothly, preparations, examinations, then to the room, lunch, then another conversation, in general, even things like showering, everything really went smoothly.

Code: ● Patient Care

IG Position: 29 - 29

So there was really always someone there whenever something came up.

Code: ● Patient Care

IG Position: 37 - 37

You probably could have done more in terms of physical activity in the hospital. So on Sunday, I knew the (second) surgery definitely wasn't going to happen, but I actually did a lot on Sunday, walked down the hallway, everything went well. But well, without food and drink, it affected my circulation.

Code: ● Patient Care

IG Position: 52 - 52

Um, yeah, I know that the difference is that I went straight to the IMC ward. That I was extubated earlier, although I can't remember that. And, um, I don't know if that's really a difference, but that I went straight from the hospital to rehab. Yeah, and otherwise -. I think that the anesthesia management is also a bit different, but in detail, I don't actually know that. And of course, then, that the whole thing, so to speak, happened more within this ERAS team. And I think they also communicated more among themselves, if I've understood that correctly. Exactly.

Code: ● Patient Care

IG Position: 10 - 10

But there was always someone there from the nursing staff and the physiotherapists, it was just as I had imagined it.

Code: ● Patient Care

IG Position: 32 - 32

I always say, I'm happy because I can keep living. And everything just went so well with the surgery and the care. Without the surgery, I don't know how much longer I would have lived. And now I can do everything. I can do sports. I'm starting slowly now, but I'm already getting pretty far again.

Code: ● Patient Care

IG Position: 111 - 111

This all-around care was, of course, how should I say, perfected. I was very satisfied. So it starts with the information, right from the beginning. So great.

Code: ● Patient Care Gewicht: 0

IG Position: 10 - 10

I

How was your stay in the hospital, then?

B

That really was also very positive. The ERAS nurse took really good care of me. That was very helpful, for example, that I received a nicotine patch. That actually helped me a lot. So, you can't imagine but I'm actually a non-smoker now, and I'm so proud of it. And so, I think she and the [psychosomatic specialist] really helped me with tips for quitting smoking. And then of course, the physiotherapy, which was constantly there and kept me occupied. So yeah, really great. I was constantly mobile, and I think that also helped me a lot.

Code: ● Patient Care

IG Position: 24 - 26

Because I was in the ERAS group with all that information, with that whole great concept behind it. No, I really can't think of anything, there's nothing that could be improved, it was really, well, optimal, it should always be done like that. It would be nice if it could work out like that for everyone and always, that would be truly invaluable.

Code: ● Patient Care

IG Position: 97 - 97

I

Yes, all right. Um, regarding other decisions: Did you feel more like you were perhaps, well, I'd say, more taken into account there? Even though, as you just mentioned, you were also asked here.

B

Yes, that was the only critical aspect. Otherwise, I can't say anything negative about it. So, I'm not sure what you mean by decisions - you see, that was the only decision where I say, "Hm, that was a bit premature."

Code: ● Patient Care > Involvement in Treatment Gewicht: 0

IG Position: 95 - 97

I

Um, then let's continue. Did you feel involved in the care process, I mean, in your treatment, as well? Did you feel like decisions were made together with you?

B

Whether decisions were made together with me - well, beforehand, it was discussed what needed to be done or should be done. There was also a discussion about - if the valve needed to be replaced, which was the case - which one I preferred. The different options were presented to me again. Well, then I went into surgery and I just handed over control. And where I say, yes - and afterwards, it was actually explained what was done, although I don't remember everything because I was still a bit groggy from the anesthesia, but nevertheless, I was told again what was done. And in the days following, there were regular visits where information was provided. Information also came from the ERAS team.

Code: ● Patient Care > Involvement in Treatment Gewicht: 0

IG Position: 84 - 86

I

Exactly, so you mentioned earlier that the care process - the joint decision-making - was more at the beginning. But how did you experience the exchange of information during the process?

B

That was good. So, I can't complain. If I wanted to know something, then I got the information, or sometimes the information just came. If there was a ward round or something, yes. No, it was fine.

Code: ● Patient Care > Involvement in Treatment Gewicht: 0

IG Position: 92 - 94

Ultimately, it was also the kind efforts of the ERAS team that made a big difference. So, I can only commend them for that. And generally, the nursing staff provided quite decent care as well. Yes, and otherwise, well, they conducted their ward rounds, they checked in, or something like that. And before, I was properly informed by various doctors about what was going on and what needed to be done during the surgery. I was adequately briefed beforehand. And afterward, from a medical standpoint, well, they conducted their rounds, and when complications arose, I was also properly informed, and then things moved swiftly, yes. So, I can't really say that decisions were made over my head during or after the surgeries - it was simply dictated by the situation, and accordingly, actions had to be taken, yes. (I: Okay.) So, I see it quite pragmatically in that regard, yes. One could also have said, "There are complications, well, I don't want to be operated on." But there wasn't really a major decision to be made, yes.

Code: ● Patient Care > Involvement in Treatment Gewicht: 0

IG Position: 118 - 119

I

Okay. Then again, regarding the process of your care, so your entire treatment actually, did you feel well integrated there?

B

Certainly, yes, because I actually always had someone to talk to and really had the opportunity, whether it was from the nursing staff or a senior physician – whatever. So I was also able to ask questions, so I never felt like I was somehow, yes, practically not noticed. I could always contribute my part in some way.

Code: ● Patient Care > Involvement in Treatment Gewicht: 0

IG Position: 72 - 74

I

Yes, then could you also participate in or at least did you feel like you were making decisions and having a say in how things would proceed for you?

B

Yes, because everyone said: "Always to the extent that suits me." I am the one it concerns, I practically have to say, what do I want, what can I do. So even if someone told me: "You're going to do this now, you can do it." But if it wasn't possible, then it wasn't possible, because I am the one, yes, my body, so I could always and at all times determine what I wanted.

Code: ● Patient Care > Involvement in Treatment Gewicht: 0

IG Position: 80 - 82

I

Did you feel involved throughout the entire process? Did you generally feel included?

B

Yes. Definitely, yes.

I

Okay, are there any specific examples?

B

Yes, in terms of feeling involved, I was already engaged because I always had those questionnaires and those daily reports. So, this diary that I had to write, yes, I felt really involved with that, I liked that, yes.

Code: ● Patient Care > Involvement in Treatment Gewicht: 0

IG Position: 60 – 67

B

But except for Corona, otherwise, I couldn't have wished for it any better. You're not just a stranger in the hospital, but you're a patient. And it's clear to the others what's happening to you, why you're even there, And that this is also something big for the patient. That made me very happy, and I wish it would always be like that in the hospital and rehab.

Code: ● Patient Care > Involvement in Treatment Gewicht: 0
IG Position: 107 - 107

Yes, enough. My ERAS nurse, in particular, gave me a lot of information. She always took good care of me. She took a lot of time for me. And she was always with me. I could always turn to her. But also the doctors and the professor were with me. I felt well cared for. But I was only in the hospital for 5 days. And then I went directly to rehab. Everything was organized for me, and that was great.

Code: ● Patient Care > Involvement in Treatment Gewicht: 0
IG Position: 91 - 91

I

Did you feel involved in your care process?

B

Yes, that was completely sufficient, I always knew exactly what was going on. So, all good. Yes.

Code: ● Patient Care > Involvement in Treatment Gewicht: 0
IG Position: 36 - 38

I

Yes, did you also have any further conversations, for example?

B

Actually, before the surgery, the psychosomatics lady called me again because I was quite nervous.

I

Actually, before the surgery, the psychosomatics lady called me again because I was quite nervous.

B

Well, more like, uh, yes, more of a disaster - I don't know what to say there. I really felt bad at times.

I

Yes.

B

So I also sought help from an additional therapist - my psychologist - no, he's not a psychologist, he's a therapist. And, well, my condition before the surgery was really, really bad - not just physically, probably more mentally than physically.

Code: ● Patient Care > Mental Health / Support Gewicht: 0
IG Position: 39 - 49

I

Okay, um, did you then, in the process, when you were ultimately undergoing treatment, still feel safe or well taken care of? Or did the stressful situation, I mean, overshadow everything?

B: Yes, well, I did feel well taken care of, but at the same time, after the surgery, I was severely weakened by my pneumonia. So that was really, uh, a bad mix, yes. It really weakened me and also burdened me.

I: Yes, um, when you compare that now: How were you feeling psychologically after the surgery back then?

B: Yes, the question is - are you asking when after the surgery? Whether three weeks, three months - that's a difference.

I: Yes. Then I would just start directly, so if you now mentioned a time directly three weeks after the surgery?

B: Three weeks after the surgery, I still felt very bad psychologically.

I: Okay, yes. Do you have a point in time where the situation improved - or is it still ongoing?

B: So psychologically, it got better after the rehab, that's when I started feeling better, and it's been steadily improving since then.

I: Okay, very good. Looking back on it now - especially because it represented a great burden for you and still does - did you feel that the surgery was still the right decision?

B: Absolutely, yes. From today's perspective, I am very grateful - also for the course of the surgery. Um, but I have to say it was a difficult journey, yes.

Code: ● Patient Care > Mental Health / Support Gewicht: 0
IG Position: 51 - 69

Yes, for quite some time before the surgery, I have to say, I was actually doing quite well. Of course, one keeps thinking: "Why me? Why now? Where does this illness practically come from?" - etc. those kinds of things. And the closer the whole thing naturally got, the date, the more, well, the more the excitement naturally increased a bit, and somehow a bit of an uneasy feeling - you probably always have that before a major surgery. And, but for me, it was also the first time something like this, or such a big deal - I had never experienced anything like it before. And at some point, you start counting the days. Yes, it's like a countdown, which then runs out, and of course, you get a little more tense with it, yes. And that was one side of the coin, then after the surgery - on the day after the surgery, actually, yes, within the realm of possibility, actually a good feeling, you would have thought it would be quick, but then there was a setback. So, physically then as well, then logically also mentally, it takes quite a toll on you, and then in the end, for me - I'll say - that lasted about two, three days, then things started to improve - I was still in the hospital.

Code: ● Patient Care > Mental Health / Support Gewicht: 0
IG Position: 46 - 46

I was then transferred to a rehabilitation facility where I said, 'Let's step on the gas and get me back to full health.' However, I experienced another episode of health issues, specifically water on the heart and atrial fibrillation. As a result, I was transferred back to the hospital, which set me back at least a week in terms of physical fitness. This was also a psychological shock. During my time in rehab, I had to deal with the fear of experiencing another episode of atrial fibrillation or water-related issues, which could result in hospitalization. This fear persisted even after leaving rehab and caused me significant stress. It took me some time to come to terms with this fear.

Code: ● Patient Care > Mental Health / Support Gewicht: 0

IG Position: 46 - 46

I

During this time, did you feel that you were in good hands with the staff or the ERAS process, despite any anxiety or justified concerns you may have had?

B

Yes both the psychosomatic nurse and the ERAS nurses were there almost every day during my first hospital stay and also when I was transferred back [to the hospital] the second time, um - I don't know how often they were there - but they looked after me, talked to me, discussed various things and gave me courage. So that was very good. Especially when you might have had a low point or something, where they helped you overcome it, gave you courage, and straightened you up.

Code: ● Patient Care > Mental Health / Support Gewicht: 0

IG Position: 70 - 70

I

Was the psychosomatic aspect also helpful in the education and care provided by ERAS?

B

During a discussion on this topic, a woman from psychosomatics warned me in advance about the possibility of experiencing low points. She advised me to be careful and not let them discourage me. Although these low points are common, they can still be difficult to deal with when they occur, even with the knowledge that they are likely to happen. As previously mentioned, they were present in every case, including the aftermath, and provided assistance. I need to reflect on this further.

Code: ● Betreuung > Mental Health / Support Gewicht: 0

IG Position: 72 - 74

As previously mentioned, my wife contacted the psychosomatic department regarding the complications during rehab, specifically related to water on the heart or atrial fibrillation. This was an emotionally challenging phase, culminating in a transfer back to Augsburg during the night. It was a nerve-wracking experience for everyone involved.

Code: ● Patient Care > Mental Health / Support Gewicht: 0

IG Position: 74 - 74

Psychologically, it was challenging, but ultimately clarifying. However, it required careful digestion.

Code: ● Patient Care > Mental Health / Support Gewicht: 0

IG Position: 42 - 42

I

Okay, yes. Then let's move on to the next question, how were you feeling mentally or psychologically - both before and after the surgery?

B

Before the surgery, I always felt like I was living with a sword of Damocles hanging over my head. It was a difficult time for both of us. The constant uncertainty of whether something would happen or not was a great source of stress, not just for me but also for my sister, who I am very close to. After the surgery, complications persisted, requiring a second surgery. This was the lowest point. However, once everything was sorted out in the clinic, the worst was over. With caution, things slowly improved.

Code: ● Patient Care > Mental Health / Support Gewicht: 0

IG Position: 60 - 62

I

Yes, I think so. How helpful was the psychosomatic care, or education, during your treatment?

B

That was also OK, it was a good fit, yes. That you know, whennever your thoughts are a bit bad, that this could also have an effect. And when you know that: "Aha, you feel like shit because it's connected." - then everything is put into perspective and you don't panic as much as you would if you didn't know where it was coming from. So, as I said, the information content was - well, I felt well informed.

Code: ● Patient Care > Mental Health / Support Gewicht: 0

IG Position: 96 - 98

And that also helps to calm the psyche a little, I'd say. And not just for me, but also for my relatives - primarily my sister, yes.

Code: ● Patient Care > Mental Health / Support Gewicht: 0

IG Position: 166 - 166

I

Okay, you just said that you were basically afraid of this surgery. But did the team that accompanied you give you a sense of security?

B

Yes

Code: ● Patient Care > Mental Health / Support Gewicht: 0

IG Position: 44 - 46

Okay, so how was it with the contact person for psychosomatics and the information about it?

B

That was also great because, as I said, I already had - or am a bit unstable in psychological matters. The lady [from the psychosomatic specialist] gave me a lot of information beforehand and practically

took care that I was psychologically cared for in the rehab. Therefore, that was a bit of a red thread running through it. And so it was actually a bit of a common thread that ran through - in that sense.

Code: ● Patient Care > Mental Health / Support   Gewicht: 0

IG   Position: 60 - 62

The fixed date of my surgery was crucial to me as I was naturally excited and nervous beforehand. The fixed date of my surgery was crucial to me as I was naturally excited and nervous beforehand. The fixed date of my surgery was crucial to me as I was naturally excited and nervous beforehand. During the time of my surgery, there was a strike, and I had heard that many surgeries were being postponed. I have spoken to other patients in rehab who were sent home for a fortnight and then had their appointments postponed again. I recall that the given date for my surgery was honoured, which relieved me of the anxiety of being sent home due to unavailability. This was a great relief for me.

Code: ● Patient Care > Mental Health / Support   Gewicht: 0

IG   Position: 158 - 158

In the beginning, I had real psychological problems with this surgery. Yes, but it really was better after the explanatory talk. I thought that was very good, yes. There was a familiar face there immediately after the surgery. I was really well taken care of.

Code: ● Patient Care > Mental Health / Support   Gewicht: 0

IG   Position: 23 - 23

So I found the psychosomatic counselling good, yes. Yes, very satisfying. Above all, the whole thing was somehow very calming.

Code: ● Patient Care > Mental Health / Support   Gewicht: 0

IG   Position: 39 - 39

I found the psychological counselling helpful. Yes, then the information from the operating theatre about what was being done. Yes, the information about the ERAS, what's there. The aftercare and the whole thing, I thought that was good. It was really helpful.

Code: ● Patient Care euung > Mental Health / Support   Gewicht: 0

IG   Position: 43 - 43

The most useful thing for me was the discussion with psychosomatics, i.e., how the mental course could be. That there can be lows after the surgery. But this is also quite normal, and it usually gets better continuously. And also to see the whole thing in a positive light.

Code: ● Patient Care > Mental Health / Support   Gewicht: 0

IG   Position: 42 - 42

Knowing who you can come back to and having a fixed contact person helped me a lot.

Code: ● Patient Care > Mental Health / Support   Gewicht: 0

IG   Position: 58 - 58

There was always someone there if anything happened. I could always ask someone. That was really good too.

Code: ● Patient Care > Mental Health / Support   Gewicht: 0

IG   Position: 67 - 67

Throughout the whole process, “I didn't just feel like a number but as a patient being treated and cared for. And that is something that was very important and good for me. I had the feeling that I was being no-ticed. Not just as another patient, but as a person. Both in hospital and at the rehab centre.

Code: ● Patient Care > Mental Health / Support   Gewicht: 0

IG   Position: 87 - 87

So I think ERAS, the way I experienced it, is a great help for me. For my recovery, for my mindset.

Code: ● Patient Care > Mental Health / Support   Gewicht: 0

IG   Position: 107 - 107

But apart from that, I couldn't have wished for anything better. That you're not just a stranger in the hospital, but that you're a patient and that the others realise what's happening to you, why you're there in the first place and that it's also something big for the patient. I was really happy about that and I wish it was always like that in hospital and in rehab.

Code: ● Patient Care > Mental Health / Support   Gewicht: 0

IG   Position: 107 - 107

So then you also had contact with the ERAS nurse, how did you find her?

B

Well, I think that was just a huge support for me. You just didn't feel so alone. And if you had a question, you could clarify it with her straight away.

Code: ● Patient Care > Mental Health / Support   Gewicht: 0

IG   Position: 52 - 54

So looking back, now that it's all over, you think to yourself "It wasn't all that bad after all", but in the run-up to it, yes, it's a huge strain, especially psychologically.

Code: ● Patient Care > Mental Health / Support   Gewicht: 0

IG   Position: 82 - 82

No, that was enough. But then you just ask yourself whether you're really covered enough if you don't wake up. Even if the risk of something worse happening was rather low. But it's a strange feeling for such a layman. When you imagine lying there on the operating table and your heart is no longer beating. (Short pause)

Code: ● Patient Care > Mental Health / Support   Gewicht: 0

IG   Position: 86 - 86

How did you perceive the ERAS nurse?

B

Well, it just feels good. It's really good to have this support.

I

Can you explain why?

B

Yes, because you always have someone there. And if you have a problem, you already know them. And with nurses you don't know, you don't say anything, but you have someone you can talk to about it.

Code: ● Patient Care > Mental Health / Support Gewicht: 0

IG Position: 70 - 76

That was good, good preparation. It was really good. I was really scared of the surgery. But this preparation really helped me. The psychological aspect too. And I was then able to contact the woman [psychosomatist] again before the surgery. That was also good because I was sometimes very scared. That calmed me down again.

Code: ● Patient Care > Mental Health / Support Gewicht: 0

IG Position: 14 - 14

The psychosomatic counselling and the preliminary discussion definitely eased my fear of the surgery.

Code: ● Patient Care > Mental Health / Support Gewicht: 0

IG Position: 25 - 25

Exactly, yes, it's actually the case that the conversation was simply reassuring for me now, that I just know that a lot of people know about it, or that I have some people I can approach afterwards for various things if something should happen. That was good.

Code: ● Patient Care > Mental Health / Support Gewicht: 0

IG Position: 44 - 44

And what I also found good [in the preliminary psychosomatic consultation] was that we made a list like this. What could occur, like pain, fear, whatever. And then, we always tried to find possible solutions or behaviors that could be used to counteract this. That helped me most of the time. Well, not always, but it was good and, above all, she] had named some things, where I would not have thought about at that moment, that this could happen. And in this respect, that was very good.

Code: ● Patient Care > Mental Health / Support Gewicht: 0

IG Position: 49 - 49

As I said, the preliminary discussion was of course good. I thought the whole process was very clear. In terms of the procedure, I wouldn't say it was very surprising for me. It was simply clear, first a preliminary consultation, then surgery, then rehab. So everything was actually already clear beforehand. And as I said, it was quite good for me, like when you're lying there after the surgery and are told that we first have to see when a rehab place becomes available. This predetermined structure was helpful for me.

ode: ● Patient Care > Mental Health / Support Gewicht: 0

IG Position: 103 - 103

Yes, definitely. It was really good to know that someone was looking after you and that you wouldn't get lost in this big hospital. That's a really big plus.

Code: ● Patient Care > Mental Health / Support Gewicht: 0

IG Position: 59 - 59

I was able to accept the idea of needing heart surgery at a young age calmly. Being well-informed before the surgery helped me approach it with peace of mind. It is possible that people in the other group were not as relaxed as I was. I was well-prepared and informed about everything that was coming my way.

Code: ● Patient Care > Mental Health / Support Gewicht: 0

IG Position: 18 - 18

I was aware that a physiotherapist would be present next to me shortly after the surgery, and would assist me in standing up and so on. This information was very helpful to me.

Code: ● Patient Care > Physiotherapy Gewicht: 0

IG Position: 101 - 101

I

During your treatment, did anything improve or worsen your acceptance? For instance, did the physiotherapy, which was carried out soon after the surgery, have any impact on your mood or cause you to reject it?

B

It is positive that someone is present every day to encourage and motivate individuals to get up and start their day. However, there were times when I was not feeling well and unable to participate. However, there were times when I was not feeling well and unable to participate. Overall, I appreciate the daily support and care provided.

Code: ● Patient Care > Physiotherapy Gewicht: 0

IG Position: 158 - 161

Afterwards [after the surgery] it was helpful - so I can really only recommend the ERAS programme, a heart surgery like that is a big break in life, er, and a big box and the more information you have, the better. And also the programme afterwards, with only intensive care for a short time and, er, with - with all the animations by the physio. I think the concept definitely helps people to get fit again more quickly.

Code: ● Patient Care > Physiotherapy Gewicht: 0

IG Position: 213 - 213

On the one hand, I thought it was good that the physio was a little more intense than the normal stuff - that helped me to get on my feet a little more, of course, than if I hadn't had that - the more intensive physio.

Code: ● Patient Care > Physiotherapy Gewicht: 0

IG Position: 94 - 94

The physiotherapy sessions were more extensive than usual, which was convenient for me. Subjectively, I feel that it made me a few percentage points fitter than if I had not attended the sessions. Additionally, the presence of the physiotherapist motivated me to continue with my exercises.

Code: ● Patient Care > Physiotherapy Gewicht: 0

IG Position: 166 - 166

I

Okay, was there a person or perhaps a professional group that helped you in particular?

B

Yes, the nursing staff in particular - the physiotherapists too, to be honest. It's an advantage when you're accompanied by the physiotherapists from, yes, you could say, the second hour after the surgery or the third. Where you might be unsure yourself - "What am I allowed to do now?" or "Am I allowed to walk, stand up, whatever?" Where they have always practically encouraged you to do it, yes and, or you have dared to do it.

Code: ● Patient Care > Physiotherapy Gewicht: 0

IG Position: 76 - 78

I

Okay wonderful, then you have answered this question - really all the sub-questions - too. Then again about the treatment process and your care: would you say that this whole process - also with ERAS - has contributed to your, yes, own health again?

B

In any case, I have to say that it was a challenge to get up quickly and have physio straight away. However, it is undoubtedly pleasant when you can go to the toilet independently the same morning or the next day, and the urinary catheter has already been removed. You are no longer tied to your bed and are physically challenged by the physio, which gets your circulation going more quickly. While it has been a challenge, I have found the recovery process to be very positive compared to other patients I have seen.

Code: ● Patient Care > Physiotherapy Gewicht: 0

IG Position: 120 - 122

So physiotherapy for me, in hospital, that wasn't strenuous. Not strenuous is perhaps the wrong word, because you can't do that much at the beginning.

Code: ● Patient Care > Physiotherapy Gewicht: 0

IG Position: 62 - 62

During my first attempt at cycling, I faced several challenges such as poor oxygen saturation and difficulty maintaining an upright posture. Despite being encouraged to stand up straight and broaden my shoulders, I struggled to do so. Despite being encouraged to stand up straight and broaden my shoulders, I struggled to do so.

During my first few metres down the corridor, I struggled to continue. However, during my second physiotherapy session, I was accompanied by someone else, which made the experience more enjoyable. Overall, I was satisfied with my progress as we continued to walk along the corridor. After that, things improved rapidly, including cycling and other activities.

Code: ● Patient Care > Physiotherapy Gewicht: 0

IG Position: 110 - 115

And a physiotherapist. He came round, helped me out of bed and then we walked a few steps. So yes, I got moving again in no time at all.

Code: ● Patient Care > Physiotherapy Gewicht: 0

IG Position: 12 - 12

I believe that the sooner you get out of bed and start moving, the less weight you will gain. If you remain in bed for an extended period and then attempt to walk, it will undoubtedly be more challenging.

Code: ● Patient Care > Physiotherapy Gewicht: 0

IG Position: 20 - 20

I would say, it worked out fine. At the beginning, I might have said otherwise, but eventually, you could move around better and so on. Firstly, your mindset changes completely; you have more drive to get out of there when you're not just lying in bed, yes. So, it's actually good that they came in like that. But yeah, at the beginning, it's not exactly what you want. But I think that's quite normal. (B laughs)

Code: ● Patient Care > Physiotherapy Gewicht: 0

IG Position: 132 - 132

My main thought was actually just to get out of bed as soon as possible and get back on my feet. And from that perspective, any decision was fine with me.

Code: ● Patient Care > Physiotherapy Gewicht: 0

IG Position: 136 - 136

I

Did you also have physiotherapy? Do you remember when you had it and how beneficial it was?

B

Still in the IMC. The colleague came by immediately and got me to the edge of the bed or helped me sit up somehow. So, it started right away, or relatively quickly. I was still half-awake at the time, and I think he came by twice, then it continued the next day.

I

Was it good for you that it started so early, or was it overwhelming?

B

I thought it was good, yes. Because when I was on the ward, I had a bit of trouble getting moving. I mean, I thought I should get up, but somehow nothing happened. It's hard to describe if you haven't experienced it before, but it was always good when someone came in and said, "Okay, let's get you out of here and see if you can walk in the hallway.“ It's just that it was also quite a good motivation and support to overcome this sluggishness and get going again.

Code: ● Patient Care > Physiotherapy Gewicht: 0

IG Position: 77 - 83

Yes, every day. That was very good, so much physiotherapy. I was able to walk stairs in the hospital again. And then of course, the physiotherapy, which was constantly there and keeping me busy. So yeah, really great. I was constantly mobile, and I think that helped me a lot.

Code: ● Patient Care > Physiotherapy Gewicht: 0

IG Position: 83 - 83

And of course, the physiotherapy was always there and kept me busy. So yeah, really great. I was constantly mobile, and I think that helped me a lot.

Code: ● Patient Care > Physiotherapy Gewicht: 0

IG Position: 26 - 26

Yes, I was mobilized in bed on the day of the surgery, so I sat up and took a breath, I can still remember that, yes.

B

How was that was for you??

I

Well, I found it good, of course, I was still sleepy on the first day, but I still found it good.

Code: ● Patient Care > Physiotherapy Gewicht: 0

IG Position: 88 - 92

And if something comes up, if you're not feeling well or if you need something, having the nurses there to help you is the most important thing.

Code: ● Patient Care > Unterstützung Gewicht: 0

IG Position: 82 - 82

Especially helpful for me was the pre-op preparation: essentially, what can I do to optimize the surgical process - with nutrition, movement, exercises. I found that helpful.

Code: ● Preop Counseling Gewicht: 0

IG Position: 33 - 33

Actually, psychologist called me again before the surgery because I was very nervous.

Code: ● Mental Health Gewicht: 0

IG Position: 41 - 41

Yes, there were many things involved. Firstly, of course, the preparation for the surgery for me - in other words, what else I can do for my fitness beforehand. Some of that was already mentioned in the handbook, but there were, of course, other things beyond that. Then I also received some dietary supplements, also for fitness or preparation. And many other topics related to what to consider, what particularities there are, and how to proceed afterward, what to watch out for afterward.

Code: ● Preop Counseling Gewicht: 0

IG Position: 30 - 30

And all of this happened beforehand, and the conversation just confirmed and reassured me that, regarding the measures we had already started independently of the heart issue, I was on the right track.

Code: ● Preop Counseling Gewicht: 0

IG Position: 54 - 54

And I also had time to prepare myself. So it worked out for me, I would say.

Code: ● Preop Counseling Gewicht: 0

IG Position: 34 - 34

Yes, I found it good. I also found it good in the sense that different professional groups were actually there, so you got to know the people a bit and you already received quite a bit of explanation in advance about what would happen, how things would proceed. And one, or I also knew then, that you would see these people again, that they would come back to you when the surgery was scheduled. That's something that makes you feel relatively well taken care of, especially in such a large university hospital. So you know, okay, they already know about it and then, yes, you actually feel quite good.

Code: ● Preop Counseling Gewicht: 0

IG Position: 18 - 18

And I also had time to prepare myself. So it worked out for me, I would say.

Code: ● Preop Counseling Gewicht: 0

IG Position: 27 - 27

Exactly, we actually did an exercise with auto-suggestion, which wasn't completely new to me. But it was simply good to do it again just before, and also to have it as your own tool, to have it in your mind. And what I also liked about it was that we made a list of things that could happen, like pain, fear, whatever, and then we always tried to find possible solutions or behaviors to deal with it. That usually helped me. Well, not always, but it was good, and especially she mentioned some things or said things that I wouldn't have thought of in that moment, that could happen. So in that sense, it was really very good.

Code: ● Preop Counseling / Mental Health Gewicht: 0

IG Position: 49 - 49

That was definitely, uh, because at least for me, the amount of information - being well informed - is always very important.

Code: ● Information Gewicht: 0

IG Position: 166 - 166

Yeah, well, of course, you do consult Dr. Google sometimes. (I: Yes.) Which happens all the time. But I did that more before the conversation, actually not so much after the conversation.

Code: ● Information Gewicht: 0

IG Position: 38 - 38

I

Very good. Um, then let's move on to the information. How did you find the flow of information in the hospital? I mean, did you know at all times what was going to happen next for you and what the next steps were?

B

So that actually came across quite well. I mean, except for a few times, but well, you're not the only patient on the ward, where you just have to ask again sometimes. But it was - well, I didn't feel like something needed to be improved. Well, sometimes the coordination may not have been so good, where something, whatever, got served on the table and then, at the same time, someone came to take you in a wheelchair for some examination. Well, okay, now my meal was just standing there. But these are coordination issues where, perhaps from a procedural point of view, there really was no other way. But those are minor things.

Code: ● Information Gewicht: 0

IG Position: 84 - 86

I

Okay, and when it comes to these specific pieces of information, who mainly communicated them to you?

B

Information - actually the nursing staff, then the ERAS ladies who were always there. And yes, then of course there were doctors as well, where you also have to do something yourself or ask what was operated on.

Code: ● Information Gewicht: 0

IG Position: 88 - 90

Yes, well, they usually came and explained what was going to happen right away. Uh, so yes, that was communicated. Sort of, what happens now. And also, as I walked down the hallway, someone would come up and help.

Code: ● Information Gewicht: 0

IG Position: 75 - 75

Yes. So the transparency contributed to the fact that I already knew, "Okay, tomorrow at eleven this and that is scheduled." I'm not used to that. I also participated in a study, it was a one-hour MRI, and I always knew, there's also the physio assessment or something. Or he'll do this and that at such and such a time. And I found that very pleasant. You could also very well adjust yourself afterwards and prepare for what's going to happen next. Because otherwise, it just starts and you don't know what you're going to do next. (I: Okay.) That was very pleasant in Augsburg.

Code: ● Information Gewicht: 0

IG Position: 50 - 50

I

Okay. So did you also receive further information during your hospital stay? How was the flow of information? Did new information also come to you?

B

I can't think of anything now. So I don't remember receiving more information where I would have wanted or needed it.

Code: ● Information Gewicht: 0

IG Position: 100 - 102

Well, I usually just asked the nurse. But otherwise, you could ask anyone when you were walking down the hallway.

Code: ● Information Gewicht: 0

IG Position: 68 - 68

I

Did you then receive all the information you wanted or needed?

B

I would say so.

I

Okay. During your stay, did you also receive new information?

B

Yes, indeed.

Code: ● Information Gewicht: 0

IG Position: 114 - 120

A nurse or the doctor would come by. Sometimes you just asked. Quite different. Very, very different.

Code: ● Information Gewicht: 0

IG Position: 124 - 124

I

Yes, who mainly communicated these pieces of information to you - which you found helpful?

B

That was mostly, uh, my contacts from ERAS. They were my interface and where I got all the information.

Code: ● Information > Contact Person Gewicht: 0

IG Position: 103 - 105

I

Okay, understood. Did you then know at all times in the hospital whom to turn to for questions or uncertainties?

B

Yes, both doctors and, uh, assistants as well as ERAS - I always knew who to turn to, yes.

I

Okay, did you also have a main point of contact in the whole process for yourself?

B

Yes, well, I always had the ERAS number, I could always reach that ERAS team - so it was clear to me that they were there, yes.

I

Okay, and as a result, it was ultimately possible to clarify these questions or uncertainties or concerns of yours accordingly, yes?

B

Yes.

Code: ● Information > Contact Person Gewicht: 0

IG Position: 135 - 145

And in that regard, it was already good when, yes, almost daily at the beginning, there was always someone available as a contact person.

Code: ● Information > Contact Person Gewicht: 0

IG Position: 70 - 70

I

Okay. Who mainly communicated this information to you?

B

So mainly actually the staff from ERAS [Nurse and Psychosomatics], who then ultimately provided me with information during their daily visits - and, uh, yes. I have to say, that's where most of the information came from. Sometimes, of course, from the ward staff if there was something specific, and sometimes also from the doctors. But mostly, I would say, it was really through the ERAS team, followed by the ward staff.

Code: ● Information > Contact Person Gewicht: 0

IG Position: 96 - 98

I

Okay, understood. Then let's move on to the next question: During your treatment, did you always know who to turn to if you had questions or uncertainties?

B

You could say so, yes.

I

Okay, were there situations where you didn't know?

B

No, not really.

Code: ● Information > Contact Person Gewicht: 0

IG Position: 136 - 142

I

Okay, and if you had to phrase it now, was there someone or, yes, essentially a group of people who actually served as the main point of contact?

B

Yes, of course - because the most readily available ones were the ward staff. And as the days went by, you got to know one or the other a little better, could also differentiate a bit, who was more experienced and who wasn't, and then naturally you turned to those people.

Code: ● Information > Contact Person Gewicht: 0

IG Position: 144 - 146

I

Yes, and also the ERAS team, to what extent could they also serve as contact persons?

B

Well, because they were almost there daily or especially during the first stay, they were really there daily, I knew someone would come, and as a result, I knew if something was up, I could discuss it there. I did ask a few questions there, and they were answered or clarified relatively quickly. So, that worked well.

Code: ● Information > Contact Person Gewicht: 0

IG Position: 148 - 150

And the ERAS ladies or something like that, they were always there to help, and you were always well informed, yes. Because what I found a bit odd from other hospital stays is that you often just don't have any information, yes. Like, what happens next or something - and that was just great.

Code: ● Information > Contact Person Gewicht: 0

IG Position: 94 - 94

I

Okay. Can you recall who mainly communicated this information to you?

B

That was mainly in the preoperative discussions, the respective doctors who conducted the information sessions before the surgeries. And the ERAS ladies, so there's nothing to complain about, the information from the ERAS ladies was just great. And the doctors clarified exactly what was necessary and good. And - well, I didn't notice any deficit.

Code: ● Information > Contact Person Gewicht: 0

IG Position: 124 - 126

That was basically the local nurses at the beginning - at the ward station, or ringing the bell, yes. (I: Yes.) And if it went beyond the organizational stuff, then I also knew that the ERAS ladies would always drop by occasionally. And then I could turn to them for the more advanced things, like the rehab afterwards, directly. Uh, that was it. If it hadn't been for them, then probably someone from the social services would have taken over, yes - so I would have ultimately known where to turn to, yes. And when it comes to the very concrete things, or something like that, I'd turn to the nurses and then involve the appropriate doctors through the nurses.

Code: ● Information > Contact Person Gewicht: 0

IG Position: 154 - 154

I

Yes, with the changing staff, I have one more question about that: Did you feel that nevertheless, in this process, you had a, yes, a fixed main point of contact?

B

Uh, in the whole process, as I said, it was the two ladies from ERAS [Nurse and Psychosomatics].

Code: ● Information > Contact Person Gewicht: 0

IG Position: 156 - 158

I

Okay. So once again, regarding the process of your care, your entire treatment actually, did you feel well integrated into it?

B

Yes, indeed, because I always had someone to talk to, firstly, and I really had the opportunity, whether it was from the nursing staff or a senior physician - whatever the case. So I was also able to ask questions, so I never felt like I was somehow, yes, practically not noticed. So, I could always contribute my part.

Code: ● Information > Contact Person Gewicht: 0

IG Position: 72 - 74

I

Okay. Uh, let's continue. Did you also always know who to turn to if uncertainties or questions arose?

B

Yes, so if I didn't know what to do, or if something was unclear to me, the first point of contact was usually the ward, and if they didn't know either - for example, on the day of discharge, it was about whether I should go to rehab now, or not. Because I had problems during the night. Then, at the same time, there was somehow still the exit interview, examinations, then the physiotherapy sessions, I was supposed to complete those. Then the driver for rehab was already there. So, I must say, if I had been alone, I would have been overwhelmed. And then, as I said, another lady from ERAS came who sorted it all out for me, so that you really got a clear head.

Code: ● Information > Contact Person Gewicht: 0

IG Position: 116 - 118

So, in the hospital itself, it was actually the ERAS Nurse who was always there, the physiotherapists were always there too. The doctors, of course, the nurses. Aurea itself had the ward, the station supervisor was always there, whether it was for the next step or whatever, he was always available. The ward doctor was actually always there. And you could talk about everything. It was good, yes.

Code: ● Information > Contact Person Gewicht: 0

IG Position: 92 - 92

That was extremely helpful. Because I could always ask questions if something wasn't clear. I always got an answer.

Code: ● Information > Contact Person Gewicht: 0

IG Position: 50 - 50

Especially regarding the information and preparation before the surgery. Especially about the course of events, what exactly happens. And that you didn't feel so alone, but always had someone to explain things to you. Knowing who you could turn to and having a fixed point of contact.

Code: ● Information > Contact Person Gewicht: 0
IG Position: 58 - 58

There was always someone there when something came up. I could always ask someone. That was really good too.

Code: ● Information > Anspr Contact Person Gewicht: 0
IG Position: 67 - 67

Uh, well, I didn't know exactly. But I felt like they could all somehow help. If there was ever a question, then I would find someone to ask. But who was responsible, I didn't know.

Code: ● Information > Contact Person Gewicht: 0
IG Position: 79 - 79

So, you also had contact with the ERAS Nurse, how did you find her?

B

Well, I think she was just a huge support for me. You just didn't feel so alone. And if a question came to mind, you could immediately clarify it with her.

Code: ● Information > Contact Person Gewicht: 0
IG Position: 52 - 54

Okay. Was there a person who mainly communicated information to you? Or was it always different?

B

Yes, mainly it was the ERAS Nurse.

Code: ● Information > Contact Person Gewicht: 0
IG Position: 104 - 106

I

Alright. So, did you always know who your point of contact was if you needed help?

B

Well, it always worked by phone. With the ERAS Nurse, anytime. But, that's also over now. So, everything worked out.

Code: ● Information > Contact Person Gewicht: 0
IG Position: 126 - 128

Well, someone was always there, the ERAS Nurse, the nursing staff, the psychosomatic specialist.

Code: ● Information > Contact Person Gewicht: 0
IG Position: 77 - 77

As I said, I would say the support was quite good also through the colleagues from ERAS, so the ERAS Nurse and the psychosomatic specialist. Someone was always there, you could actually always ask someone something, or ask for something. Where I'm a bit hesitant is that after the surgery, there

was never anyone there who had been there during the surgery. So, it was always some other assistant doctors. And I still had a few questions. I would say, it would have been nice, but I didn't want to make a fuss about it, and I would have found it quite nice if there was someone there who had been in the surgery and could say something about it. But there was always someone from the nursing staff and the physiotherapists, that was how I imagined it.

Code: ● Information > Contact Person Gewicht: 0

IG Position: 31 - 32

Then you mentioned earlier that you would have liked to have someone to ask questions to after the surgery. How did you otherwise perceive the flow of information? So, did you receive good information that concerned you?

B

Yes, definitely! That worked really well. It flowed.

Code: ● Information > Contact Person Gewicht: 0

IG Position: 73 - 75

Well, partly it was really to the nursing staff, especially when it came to medication or stuff like that. Yeah, otherwise I could have contacted the psychosomatic specialist or ERAS Nurse at any time, or I could have asked the nursing staff to contact them. But there was less need for that. Of course, I was happy when they were there.

Code: ● Information > Contact Person Gewicht: 0

IG Position: 91 - 91

I

Can you remember what she did for you?

B

Yes, we had a conversation before the surgery, and almost every day after the surgery, there was a lady oft he ERAS team here.

Code: ● Information > Contact Person Gewicht: 0

IG Position: 44 - 46

Information? Yes, of course, yes, definitely. The ERAS Nurse and the psychosomatic specialist helped me a lot, no matter what information it was. They were my main contacts. Especially the ERAS Nurse, of course.

Code: ● Information > Contact Person Gewicht: 0

IG Position: 68 - 68

Yes, the ERAS Nurse was assigned to me, she was already my main contact person.

Code: ● Information > Contact Person Gewicht: 0

IG Position: 84 - 84

I

Yes, wonderful. You mentioned earlier that you had an information session. Do you remember or can you recall when this information session took place?

B

That was about three weeks before the surgery, I would estimate.

I

Exactly, did you also feel adequately informed during this session or how did you perceive the conversation?

B

That was very helpful - that was a very positive day. I was well prepared, indeed.

I

Okay. Would you say that you also had the opportunity to ask your own questions during this session?

B

Yes, I had the opportunity. I was just very, let's say, anxious and didn't want to know all the details of the surgery - so I didn't ask for as many details as others might have.

I

Yes, but was consideration taken for that in the sense of: You still felt comfortable with it?

B

Yes, consideration was taken for that - everything was great.

Code: ● Information > Preop Counseling Gewicht: 0

IG Position: 15 - 29

Actually, it was during the examination back then, end of February was the first conversation. Then shortly afterwards the ERAS Nurse called me again and we discussed the individual details of ERAS, the individual options, relatively extensively. Afterwards, the doctor called me, and then about - what was it - four weeks, no, two weeks before the surgery, there was another very detailed appointment on-site, where the individual doctors, the anesthetist, a senior physician, a surgeon, a physiotherapist, etc., were present. The psychosomatic specialist was there, and so on and so forth. And that was another quite detailed appointment, so it was like a preliminary information session. (I: Alright.) That was at the end of March, yes.

I

Okay, did you feel adequately informed during the discussions - especially during this last major information session?

B

Yes, absolutely. (I: Okay.) That was several hours at once.

I

Yes, did you also have the opportunity to clarify questions that you had directly during those discussions?

B

Yes, definitely.

Code: ● Information > Preop Counseling Gewicht: 0

IG Position: 18 - 26

I Okay, you had an information session before the whole process, right?

B Exactly!

I Do you remember when that conversation took place for you - in relation to the surgery?

B Oh, that was around mid-February.

I Okay. And the surgery, I can check it right now, was at the beginning of March, correct?

B The surgery was - um - at the end of March because the surgery - because the appointment got messed up, it was postponed again.

I Okay, understood. Um, focusing on the information session, did you feel adequately informed during that?

B It was comprehensive, yes.

I

Okay, did you also have the opportunity to clarify questions during that?

B

I did. I Okay. Were there specific questions that you might still remember now - that were important for you to clarify back then? B Ah, like how the medication would look like after the surgery. (I: Yes.) What the potential specific consequences could have been depending on the heart valve - those were the things.

I

Okay. What did you find particularly helpful about this conversation?

B

That we were extensively informed - that the consequences were also clearly illuminated. Um, that was - and also how the surgery itself works, um, so that you just know what basically happens. That was very helpful.

I

Okay, when you compare that with the situation before and after the conversation: How did you feel after the conversation? Regardless of whether you already had concerns beforehand, or whether they came up with the conversation.

B

Before, everything was a bit vague, with the conversation it was somehow clear - what was going on, what the consequences are with and without surgery. Yeah, so, mentally it was quite tough - I would say. But it was somehow good clarification, but you had to digest it properly first, yeah.

I

Okay, yeah, I can understand that very well. How did you then prepare for the surgery after the conversation?

B

Um, I approached everything very calmly - that was basically it, yeah.

I

Okay, so for example in terms of diet or exercise, did you change anything?

B

I completely stopped exercising because with the aneurysm: exercise means high blood pressure, means death - quite simply.

I

Alright, understood. And regarding diet, that was surely also part of the conversation, did you adjust anything there?

B

Not much overall, because dietary changes or something like that, they always like to cause such actions. (I: Yes.) I had actually already completed that dietary change more or less beforehand because the dietary change had already happened long before - and then I said at some point, I weigh too much and that was already a long time before - and that's when the dietary change came in, yeah. To drastically reduce meat consumption, um, drastically reduce meat consumption - and then switch to good alternatives, yeah. And that happened beforehand and the conversation basically just confirmed and reassured me that concerning those measures, which we had already started independently of the heart issue, I was on the right track.

Code: ● Information > Preop Counseling Gewicht: 0

IG Position: 12 - 57

I

You also had an information session accordingly. (B: Exactly.) Can you remember when that roughly took place?

B

Well, it was relatively close to the surgery date because as an ERAS patient, I practically slipped into ERAS, it was actually two weeks before the surgery date.

I

Okay, did you feel adequately informed during that appointment?

B

It was really great that my husband was also able to be there. Because]. In such moments, you're often a bit nervous yourself or forget some of the questions. And if your partner is there, he can actually stand in for you or have other questions. So I found that very pleasant.

I

Yes, I understand. Did you also have the opportunity to ask questions during the conversation?

B

Definitely, definitely, so both my husband and I, and as I said, we had already thought about things beforehand. So there was really enough time.

I

Okay, how did you feel after the conversation then? Maybe also compared to before the conversation? When everything is probably very new and, well, somewhat scary.

B

Yes, so I have to say, it is of course a conversation, through which, because you also get told by the surgeon, what happens during the heart surgery, what happens to you. So it was actually like, after the five to six hours, on Augsburg dialect terms - "Maus-hi" (Note: a regional expression) it was. Because there are so many impressions that come at you. On the other hand, it was of course so that you knew exactly what was coming for you. So it's a bit of a two-edged sword, it's advantageous, but of course, you know exactly what can happen.

Code: ● Information > Preop Counseling Gewicht: 0

IG Position: 20 - 31

Let's put it this way, afterwards, the whole - that, the whole information session was done by the surgeon or the anesthesiologist. You then thought, oh my God. Through the most precise, conscientious information *I often thought afterward, "Boah, I didn't want to know exactly where the tubes come out and what can happen.* But on the other hand, it's of course also great, but it's a bit of a feeling where you think: "Hmm, yes, I didn't want to know that in so much detail." It was very exhausting

Code: ● Information > Preop Counseling Gewicht: 0

IG Position: 66 - 66

Very good. At the beginning, I really had problems with this surgery. Yes, so, somehow psychologically. Yes, but after this information session, it was really better, yes. I found that very good, yes. There was a familiar face there immediately after the surgery. I was really well taken care of.

Code: ● Information > Preop Counseling Gewicht: 0

IG Position: 23 - 23

I

Okay. And did you then have enough opportunities to ask questions as well?

B

Yes, there was enough time.

I

Okay. Did you then feel afterwards that you were informed about everything?

B

Yes. Absolutely.

Code: ● Information > Preop Counseling Gewicht: 0

IG Position: 25 - 31

That was very helpful. Both for me and for my wife. I was really glad about that.

Code: ● Information > Preop Counseling Gewicht: 0

IG Position: 22 - 22

B

As long as the person was there. There were different people there, with whom the conversations took place, from the different areas. And there you could ask everything, the conversation also lasted quite long, until you were satisfied.

I

So were you satisfied afterwards?

B

Yes. Yes. Definitely.

Code: ● Information > Preop Counseling Gewicht: 0

IG Position: 34 - 38

The most helpful thing, well, there were a few. That with the psychosomatics, like how the course could be. That there can also be lows after the surgery, but that this is also quite normal. That it usually gets better continuously. But also to see the whole thing positively. And then also the surgery, or the process of the surgery, let's put it that way, how one can imagine it. But actually, I have to say, all the conversations were really good and very helpful. And also very enlightening. So in the end, there was nothing left where I still had questions.

Code: ● Information > Preop Counseling Gewicht: 0

IG Position: 42 - 42

The information and preparation before the surgery [helped me]. Especially the course of events, what actually happens. And you didn't stand there alone but always had someone to explain things to you. You knew whom you could return to and had a fixed contact person.

Code: ● Information > Preop Counseling Gewicht: 0

IG Position: 58 - 58

Yes, well, I was nervous, of course. So that was the day when all the conversations took place. They were all very informative and enlightening.

Code: ● Information > Preop Counseling Gewicht: 0

IG Position: 30 - 30

I

Okay. Did you then also have enough opportunity to ask questions?

B

Yes.

Code: ● Information > Preop Counseling Gewicht: 0

IG Position: 40 - 42

I

Okay. Then you also had the information session a month before the surgery. (B: Yes.) How was that for you?

B

Well, very comprehensive. (B laughs)

I

Because it was so long? Or what was it about?

B

Yes, it was just an incredible amount of information.

I

Could you absorb all the information?

B

Hmm, well. Probably not everything, but mostly.

I

Were you alone at the information session?

B

My son was there.

I

Okay. Great. Did you have enough opportunity to ask your own questions?

B

Yes, definitely. I Did you also have many questions beforehand that you wanted to clarify?

B

Partly, partly.Code: ● Information > Preop Counseling Gewicht: 0

IG Position: 46 - 52

That was good, good preparation. It really helped. I was really scared of the surgery. But that preparation really helped me a lot. Psychologically too. And I was able to contact the lady [Psychosomatic specialist] again before the surgery. That was good too, because I was sometimes very afraid. That reassured me again.

Code: ● Information > Preop Counseling Gewicht: 0

IG Position: 14 - 14

I

Did you feel informed about what would happen to you later in the hospital?

B

Definitely.

I

And did you also have enough opportunities to ask questions?

B

Yes.

I

What can you best remember from that day? So which conversation stayed with you the most?

B

Well, there was a lot, generally the preparations... But directly, there wasn't anything that stuck out to me or that I specifically noticed. So it really helped, good information and that preparation. I was terrified of the surgery. But this preparation helped me well. The psychological stuff, too. And then, I could contact the psychosomatics specialist again before the surgery. That was also good because sometimes I was petrified. That calmed me down again. In any case, the preliminary education also reduced my fear of the operation.

Code: ● Information > Preop Counseling Gewicht: 0

IG Position: 16 - 25

I

Uh, then you had an information session. Do you still remember how long before the surgery that was?

B

That was, I think, about three weeks, or four weeks, I think three weeks.

I

It doesn't have to be that exact. Three to four weeks is a good estimate. Did you find the information session good then?

Code: ● Information > Preop Counseling Gewicht: 0

IG Position: 12 – 16

B

Yes, I found it good. Yes, I also found it good in the sense that different professional groups were actually there, you got to know the people a bit, and you already received quite a lot of explanation beforehand. What happens, how it will proceed. And you, or I also knew that you would see these people again, that they would come to you again when the surgery is done. That's something where you feel relatively well taken care of, especially in such a large university hospital. So you know, okay, they know it already and then, yes, you actually feel quite good.

I

Yes. Did you then also have enough opportunities to ask questions? Or did you -.

B

Yes. Definitely. Also especially with the doctor who discussed the surgery and yes. So everything that was questions at that time. (Short interruption due to construction work from until)

I

So the information session took place quite a while before the surgery. So did you find it good that it was in the weeks before, or would you have preferred a different time?

B

Hmm, well, that's a good question. So for me, it was okay. Because I was actually able to clarify the questions there. And then also had time to prepare myself. So for me, it fit, I would say.

Code: ● Information > Preop Counseling Gewicht: 0

IG Position: 18 - 27

As I said, the pre-surgery consultation was certainly good. I also thought it was good that there were different professional groups there. I got to know the people a little bit, and I got many explanations in advance about what would happen and how it would work. I found the whole process to be very clear. So, the sequence of events, I would say, was not very surprising for me. It was just clear: first the pre-surgery consultation, then the surgery, then off to rehab. So, everything was actually clear beforehand. And for me, it was quite helpful, because, you know, it's like when you're lying there after the surgery and they say, "We need to see when a spot opens up in rehab." That's something that makes me feel relatively well taken care of This predefined structure, knowing exactly what to expect, was helpful.

Code: ● Information > Preop Counseling Gewicht: 0

IG Position: 103 - 103

I

Was the information session then one day before the surgery or several weeks before the surgery?

B

Yes, a few weeks before, but I don't remember exactly.

I

Did you have, well, do you remember if you had enough opportunity to ask questions there?? Okay, so did you then feel informed afterward?.

B

I felt very informed because I had the pre-surgery consultation a few weeks before the surgery.

I

Okay. And do you remember who you spoke to there?.

B

Definitely with a doctor.

I

Did you also prepare for the surgery there?.

B

Yes, I was able to prepare for the surgery there.

Code: ● Information > Preop Counseling Gewicht: 0

IG Position: 14 - 30

So it starts right from the information session, from the beginning. Absolutely.

Code: ● Information > Preop Counseling Gewicht: 0

IG Position: 10 - 10

Yeah, well, I have to be honest, at the very beginning, I thought to myself, the less I know, the better. But in hindsight, I have to say, the whole thing really made sense. I could calmly accept the idea that I need heart surgery at such a young age. And it was precisely then, on the day of the surgery, or a few days before the surgery, that it was good that I was so well informed, because then I could approach the whole thing calmly. I can imagine that maybe people in the other group weren't as relaxed as I was at that time. And I was really well prepared and informed about everything that was coming my way.

Code: ● Information > Preop Counseling Gewicht: 0

IG Position: 18 - 18

I

Yeah. Was there anything you wished for in that process? So, something that maybe was missing?

B

Um - well, as I said, the only thing I can really report negatively is that I was discharged to rehab with pneumonia. So, I really struggled with that - I needed another week in rehab just to get started with the program. That was a bit - I found that difficult, and sometimes I wonder if it was also due to ERAS, like, "Get out of the hospital after seven days." But in my case, it was too early because of the pneumonia.

Code: ● Information > Post-OP Gewicht: 0

IG Position: 83 - 85

Um, so in the hospital, I was very - well, I was really well taken care of, so I can't say anything about that. The nurses and doctors, everyone was very friendly and, uh, also towards the patient. The only suggestion for improvement would perhaps be that the surgeon, who had the pre-surgery consultation with me, had come to the ward round again (after the surgery) and told me: "Everything's great." - I would have found that super, yes.

Code: ● Information > Post-OP Gewicht: 0

IG Position: 129 - 129

What might have been a bit of an issue was the transfer back to rehab during my second stay. There were a few, uh, how should I say, inconsistencies. First, it was said that I would stay in the university hospital until I was completely fit again, and then the next day, other assistant doctors said, "No, you should actually go back to rehab tomorrow." So, I called the attending physician and said, "That's not right." (I: Yeah.) But it was, I must say, a reasonable conversation, and the solution was for me to stay one more day - which was okay. And as I found out afterward, which I didn't know at the time, it was also important for me to return to rehab at that time because otherwise, the rehab would practically be hindered. Due to the interruption, there are deadlines because, of course, everything is somewhat bureaucratic - that's just how it is.

Code: ● Information > Post-OP Gewicht: 0

IG Position: 82 - 82

The information given to me, I think, could have been a bit better. What happened in the surgery, the whole process, and so on, there was a bit too little information. You always had to ask, ask again. And for me, since whenever I was asked, I explained or told what the situation was, what could be improved, what wasn't so good. But as for the information given to me, that could have been better.

Code: ● Information > Post-OP Gewicht: 0

IG Position: 79 - 80

Yeah, I do think I always got all the information. But I have to say, when I got the news that I needed this pacemaker, I was a bit overwhelmed at first. So what set me back was that I also needed this pacemaker. I was operated on a Wednesday. And then, they found out on Friday that I still needed the pacemaker. I was waiting all Friday and Saturday for it. Somehow, it was impossible to install it, and then I did not feel well, and there was very little communication with me. So this waiting time and the un-certainty really didn't do me any good.

Code: ● Information > Post-OP Gewicht: 0

IG Position: 73 - 73

Where I struggle a bit, so to speak is that after the surgery, there came never anyone who had been there during the surgery. So, there were always just residents. And I would have had a few more questions for the surgeon. I mean, it would have been nice, I wanted, but I didn't want to fuss about it, so I would have found it really nice if someone who was there during the surgery could have said something about it.

Code: ● Information > Post-OP Gewicht: 0

IG Position: 31 - 31

It would have been helpful if one of the doctors had directly asked if there were any further questions after the surgery. Unfortunately, this did not happen, possibly due to the changing doctors. There was also some delay in my discharge from the hospital after the surgery, as I was only able to go home one day later than expected. One doctor informed me that I could be discharged on Monday, but I expressed my concern as I was scheduled to go to rehab on Tuesday. After some discussion, the issue was resolved. However, I would have appreciated more information about the surgery and a direct update from the medical staff. They did mention that everything went well. In my case, it became apparent that I needed an additional incision during the surgery. I learned about this while in the recovery room or the IMC, while still in a groggy state. Afterward, I would have appreciated receiving more information. I will obtain this information during the final consultation.

Code: ● Information > Post-OP Gewicht: 0

IG Position: 58 - 58

No, if I wanted to know something, I got the information. So, nothing was missing.

Code: ● Information > Post-OP Gewicht: 0

IG Position: 72 - 72

Alright. Let's move on. How did you perceive the cooperation between the professional groups?

B

You mean the cooperation between the ward staff?

I

Exactly, the physiotherapy team, then of course the ERAS team, or even the medical team.

B

Well, I find that difficult to answer.

I

Yeah, I understand.

B

The ERAS staff knew about me - I don't know if they got the information from the doctors or from the ward staff - that's beyond my knowledge. Sometimes, surely, a bit more communication between doctors and ward staff would have been desirable. (I: Yeah.) I don't think everything was always coordinated. Or sometimes due to shift changes or something, but that's probably complaining on a high level. I don't know the detailed procedures, and I always have to consider - I've walked up and down the ward often enough, so there are many rooms - there are usually two patients per room, that's an incredibly large number. You can't possibly know everything about every single patient - how, how should that work, yeah. They're still humans and not computers working there. So, in that sense, I think it was okay - in terms of communication at some points, maybe it could be intensified. But yeah, as I said, there are probably worse cases than mine.

Code: ● Interdisciplinarity Gewicht: 0

IG Position: 116 - 126

I

Alright. From your perspective, were you able to distinguish who actually belongs to which professional group?

B

Yes, yes, of course.

Code: ● Interdisciplinarity Gewicht: 0

IG Position: 132 - 134

I

Okay, understood. So, how did you perceive the cooperation between the professional groups themselves? For example, did you feel that our staff members actually exchanged information about your case - and therefore, everyone was on the same page and could handle your case as efficiently as possible?

B

Well, I didn't perceive any deficiencies, as far as I could perceive anything in that direction. One is lying in the hospital room, and what happens in the background might have been good - I didn't notice anything major myself - as I said, no deficiencies perceived. They all looked well-informed - mutually. So, what may have happened or not happened beyond what I could perceive, I can't say. I can only say that whenever they were in my room - uh, in the patient's room - I didn't notice anything to complain about.

Code: ● Interdisciplinarity Gewicht: 0

IG Position: 140 - 142

So very pleasant, I must say. Whether it was the ladies from ERAS, the surgeon who stopped by, or the doctors. Then the entire nursing team, I must really say, they were courteous, friendly, and they truly addressed every wish or issue we had. So, all in all, great - truly.

Code: ● Interdisciplinarity Gewicht: 0

IG Position: 54 - 54

I

Alright. How did you find the cooperation between the professional groups themselves?

B

Well, it was very “It seemed to me that they work very hand in hand. As I said, nursing staff and then physio-therapists with ERAS ladies [ERAS nurse, psychosomatic specialist]. So everything was actu-ally already coordinated, and I had the feeling that that goes hand in hand.

I

Okay. Did you also know which employees belonged to which professional group? Was it clearly delineated?

B

Yes, well, they did - yes, the gentlemen, ladies, and gentlemen always introduced themselves as being from the physiotherapy department. Well, nursing staff, that was always recognizable. Then there was the lady from the social services, who then took over immediately, with the follow-up treatment. So, really helpful tasks were taken off one's hands.

Code: ● Interdisciplinarity Gewicht: 0

IG Position: 108 - 114

It was actually all - it went hand in hand - so it was well organized.

Code: ● Interdisciplinarity Gewicht: 0

IG Position: 154 - 154

In the hospital itself, ERAS was always there, yes, the physios were always there. The doctors, of course, the nurses. The ward itself was well-organized, the ward sister was always there, whether she comes afterwards or not, she was always available. The ward doctor was usually there too. And you could talk about anything. It was good, yes.

Code: ● Interdisciplinarity Gewicht: 0

IG Position: 92 - 92

I

So did you always know who to approach when you had questions?

B

Well, I didn't know exactly. But I had the feeling that they could all somehow help. If there was a question, I would just seek out someone. But I didn't know who was responsible for what.

Code: ● Interdisciplinarity Gewicht: 0

IG Position: 77 - 79

There were many people and they were always helpful, yes.

Code: ● Interdisciplinarity Gewicht: 0

IG Position: 62 - 62

I

Okay. Then again, when you think about your stay here, how did you perceive the collaboration between the different professional groups?

B

Well, I have to say that I didn't really perceive that. But everything went well, so it worked.

Code: ● Interdisciplinarity Gewicht: 0

IG Position: 92 - 94

I

Did you always know which staff member belonged to which professional group?

B

Yeah, pretty much. Yes, they introduced themselves accordingly if you didn't know. Yeah, that was good. (I: And -.) For example, I always had the same physiotherapist. So you got to know each other.

Code: ● Interdisciplinarity Gewicht: 0

IG Position: 96 - 98

I

Okay. Then back to the hospital staff. How did you perceive the collaboration among the staff? For example, between nurses and doctors, or physiotherapy and nursing.

B

I would say, that worked out fine.

I

So were all the information passed on?

B

Yes, I would say so. That worked out fine.

Code: ● Interdisciplinarity Gewicht: 0

IG Position: 94 - 100

I

How did you personally experience the collaboration between the professional groups, for example, between nurses and physiotherapists?

B

Quite well. The only issue was that I also had a second surgery, which made me more limited.

I

But between the different professional groups, did the work go well, especially because your situation was more complicated with the second surgery? Was the communication clear between the different professional groups, or was there initially some confusion?

B

Honestly, I didn't notice anything.

Code: ● Interdisciplinarity Gewicht: 0

IG Position: 62 - 69

Well, there was always someone there, the ERAS Nurse, the nursing staff, the psychosomatic specialist.

Code: ● Interdisciplinarity Gewicht: 0

IG Position: 77 - 77

And I believe they also exchanged information among themselves more, if I understood correctly.

Code: ● Interdisciplinarity Gewicht: 0

IG Position: 10 - 10

As I said, I would say the care was quite good, also from the colleagues from ERAS, so the ERAS Nurse and the psychosomatic specialists. There was always someone there; you could usually ask someone something or request something.

Code: ● Interdisciplinarity Gewicht: 0

IG Position: 31 - 31

I

It might also be interesting to know how you perceived the collaboration between the different professional groups. Did you notice if it went well or not so well?

B

But I couldn't really say whether they had good communication among themselves. Everyone was always in a good mood, regardless of the time of day or night.

Code: ● Interdisciplinarity Gewicht: 0

IG Position: 65 - 67

But whether they had good communication among themselves, I couldn't really say. Everyone was always in good spirits, though, no matter what time of day or night.

Code: ● Interdisciplinarity Gewicht: 0

IG Position: 71 - 71

I

How did you perceive the collaboration between the professional groups in the hospital? For example, how did you perceive the collaboration between the nursing staff and the physiotherapy staff?

B

No, everything was good, it was great.

Code: ● Interdisciplinarity Gewicht: 0

IG Position: 85 - 87

The collaboration among the doctors, the nurses, the entire staff, the social services, yes, the whole team worked together seamlessly, not to mention the physiotherapy and the psychosomatic department. So, yes, it was really great.

Code: ● Interdisciplinarity Gewicht: 0

IG Position: 14 - 14

I

How did you perceive the collaboration between the professional groups? Did anything particularly stand out to you?

B

Everything was good, everything was great, everyone was friendly, really great. I have to say, I would, I would go back to Augsburg anytime, I hope not, but I would go back.

Code: ● Interdisciplinarity Gewicht: 0

IG Position: 40 - 42

As I said, I thank everyone. It's a great team that works together, starting from the doctors, really everyone who has been involved, a super great team. Specifically, I felt very well taken care of and have already recommended it several times. So if there's ever anything wrong with your heart, go to Augsburg.

Code: ● Interdisciplinarity Gewicht: 0

IG Position: 103 - 103

I

Okay. Did you overall feel that our various staff members exchanged information about your case and therefore were all informed about how things were progressing for you?

B

I did have the impression that they knew what they were doing, yes.

Code: ● Interdisciplinarity > Communication between professions Gewicht: 0

IG Position: 131 - 133

I

Yes, I see. Do you have any further suggestions for improvement regarding the entire process, including the preoperative consultation, treatment, or rehabilitation?

B

Well, one improvement - the issue is with Marcumar. That means, you write down the INR value of two to two and a half, you suggest. (I: Yes.) And, um, the rehab center targets 3 to 3.5 - considers it correct. (laughs)

I

Ah yes, okay, so there is a somewhat differing opinion here about the values, yes.

B

Exactly, as a patient, you wonder: "What is correct?"

I

Yes, okay.

B

And Marcumar is such a big issue, especially because it's super difficult to adjust - at least for me.

I

Right.

B

The value fluctuated between 1 and 3.8 for me - I experienced everything, and I found that very exhausting.

Code: ● Interdisciplinarity > Communication between professions Gewicht: 0

IG Position: 183 - 198

Sometimes I certainly would have liked a little more communication, probably between doc-tors and ward staff.

Code: ● Interdisciplinarity > Communication between professions Gewicht: 0

IG Position: 126 - 126

As for the communication in certain areas, perhaps it could be intensified. But yes, as I said, there were probably worse cases than mine.

Code: ● Interdisciplinarity > Communication between professions Gewicht: 0

IG Position: 126 - 126

I

Yes. Um, did you find something particularly helpful or supportive during the whole process, especially in the hospital?

B

Well, what was helpful was that someone from the ERAS team was always available when I had questions. So, that was very helpful, yes.

I

Yeah. Do you have specific points where the ERAS nurse helped you?

B

Um - as I said, I talked to the psychologist once and, um - there was a visit, and then I could always ask: "Is it normal that I have this and that right now?" So that was - this support was helpful, and they were also accommodating, there were really nice people working with me.

Code: ● ERAS Nurse Gewicht: 0

IG Position: 75 - 81

I

Yes. Who mainly provided you with this information that you found helpful?

B

So mainly I got information from the ERAS nurse during her daily visits... that's where most of the information came back. There was also information from the ward staff and partly from the doctors. But most of all, I would say, really about the ERAS nurse.

Code: ● ERAS Nurse Gewicht: 0

IG Position: 103 - 105

And yes, they also partly helped me with nursing care, I can still remember when I just said: "Well, the nicest thing for me would be to take a shower again after a few days." (I: Yes.) After the surgery, because it was getting kind of creepy, you couldn't smell yourself anymore. And then they said: "Well, that's no problem, we'll help with that." Because the regular staff couldn't do it, I couldn't do it alone either, so in that sense, it was already a dream, yes.

Code: ● ERAS Nurse Gewicht: 0

IG Position: 70 - 70

And there were many other points. So, I really have to highlight the ERAS team, who were mainly my contacts for support during my hospital stay. I have to say, that was top-notch.

Code: ● ERAS Nurse Gewicht: 0

IG Position: 70 - 70

And if something happens, if you're not feeling well or if you need something, then somehow, yes, the nurses on site who help you - that's still the most important.

Code: ● ERAS Nurse Gewicht: 0

IG Position: 82 - 82

I

Okay. Who mainly provided you with this information then?

B

Well, mainly actually the ERAS staff, who then ultimately gave me information during their daily visits - and, um, yeah. I have to say, that's where most of the information came from. Partly, of course, from the nursing staff when something was up, and partly also from the doctors. But mostly, I would say, really through the ERAS team, followed by the nursing staff.

Code: ● ERAS Nurse Gewicht: 0

IG Position: 96 - 98

I

And then you came back to the hospital for your surgery appointment and also had the ERAS nurse with you. How was that for you?

B

That was extremely helpful. Because I could always ask questions if some-thing wasn't clear. I always got an answer. And you weren't alone but always had someone to explain things to you. That you knew who you could come back to and to have a fixed con-tact person.

Code: ● ERAS Nurse Gewicht: 0

IG Position: 48 - 50

Yeah. Throughout the whole thing, I didn't just feel like a number, but like a patient who is being treated and cared for. And that's already, well, for me, it was really something very important and good. I just had the feeling that I was being noticed. Not just as another patient, but as a person. Both in the hospital and during rehab.

Code: ● ERAS Nurse Gewicht: 0

IG Position: 87 - 87

I

How did you perceive the ERAS nurse? How did you find the role of the ERAS Nurse?

B

Well, she explained a lot to me during the pre-op consultation, and we discussed a lot. That was really good and helpful. Maybe to get things across.

Code: ● ERAS Nurse Gewicht: 0

IG Position: 31 - 33

In any case. Someone was there immediately after the surgery. I was really well taken care of. I was given water right away in the intensive care unit and started moving.

Code: ● ERAS Nurse Gewicht: 0

IG Position: 56 - 56

I appreciated the presence of various professional groups during my experience. Overall, I felt well taken care of and reassured that the medical professionals were knowledgeable and attentive. It allowed me to become familiar with the individuals involved and receive adequate explanations beforehand regarding the procedures and what to expect. Additionally, I found comfort in knowing that I would see these individuals again after the surgery was completed. This level of care is especially important in a large university hospital setting.

Code: ● ERAS Nurse Gewicht: 0

IG Position: 18 - 18

I

How did you perceive the ERAS nurse? What did you think of the role of the ERAS Nurse?

B

Well, she explained a lot to me during the pre-op consultation, and we discussed a lot. That was really good and helpful. Maybe to convey things a bit more.

Code: ● ERAS Nurse Gewicht: 0

IG Position: 38 - 40

I

Does anything else come to mind that you found particularly helpful during this conversation?

B

Especially helpful for me was the surgery preparation: factually, what can I do to optimize the surgery process - with food, with movements, with exercises? I found that helpful.

I

Yes, wonderful. If we could briefly touch on that: Did you have - you just mentioned it - but maybe more specifics on how you actually prepared for this surgery?

B

Yes, so in the diary, there were certain exercises, for the body - like movement exercises with a bottle, for example, weightlifting. Um, I took these nutritional supplements and tried to move as much as possible before the surgery.

Code: ● ERAS Nurse > OP Prep Gewicht: 0

IG Position: 31 - 37

I

Okay. Was there anything there that you found particularly helpful?

B

Yes, there were many things. Firstly, of course, the preparation for the surgery - that is, what else I could do for my fitness beforehand. This partly emerged from this manual, but there were, of course, other things beyond that. Then I also received nutritional supplements, um, also for fitness or for preparation. And many other topics related to it, what to consider, what special features there are, and how it actually affects you afterwards and what you have to pay attention to afterwards.

Code: ● ERAS Nurse > OP Prep Gewicht: 0

IG Position: 28 - 30

I

Alright. Then, you mentioned it just now, how did you prepare for the surgery? So nutrition, exercise were mentioned, but do you have anything else you could explain, how that actually looked?

B

Well, in the end, I did change my diet a bit, then came the nutritional supplements, and I practically did my daily exercise routine - that was about one and a half hours of various exercises per day, and a bit to strengthen the muscle again, because that was - I would say then - yes, not quite without in terms of movement restriction after the surgery. You still feel that partly today, there are still pains in the area where the surgery was done, and to actually strengthen other muscle groups a bit, to create relief, is of course not wrong.

Code: ● ERAS Nurse > OP Prep Gewicht: 0

IG Position: 36 - 38

I

Okay, yes, I can understand that very well. How did you then prepare for the surgery?

B

Um, I approached everything very calmly - that was it then, yes.

I

Okay, so for example in terms of nutrition or exercise, did you change anything?

B

I completely stopped exercising, because with the aneurysm: exercise means high blood pressure, means dead - quite simply.

I

Alright, I see. And regarding nutrition, that was surely also part of the conversation, did you then adjust anything there?

B

Nothing major, because dietary changes or something like that, which such actions always like to bring about. (I: Yes.) That was more or less already completed by me beforehand, yes. (I: Alright.) So at some point, I said I weigh too much and that was a long time ago - and then the dietary change came, yes. To drastically reduce meat consumption, um, drastically reduce meat consumption - and then with good alternatives, yes. And that all happened beforehand, and the conversation actually only confirmed in general, the measures that we had already started independently of the heart issue beforehand - it actually just confirmed and agreed with me that regarding that matter, I am on the right path.

Code: ● ERAS Nurse > OP Prep Gewicht: 0

IG Position: 44 - 54

Did you prepare for the surgery in any specific way?

B

Well, yes, I'm already a fairly agile person in terms of fitness. But then I actually brought along a little diary, which contains things you can still improve, things you can maybe start right after the surgery - exercises. I had supplemental nutrition, those were the small drinks that are highly calorific, and protein supplements, so that the body was already supplied with sufficient nutrients beforehand. And yes, I could also mentally prepare better.

Code: ● ERAS Nurse > OP Prep Gewicht: 0

IG Position: 32 - 34

In any case. Someone was there immediately after the surgery. I was really well taken care of. I was given water right away in the intensive care unit and started moving. That's where the preparation also helped.

Code: ● ERAS Nurse > OP Prep Gewicht: 0

IG Position: 56 - 56

And then I also had time to prepare. So for me, it fit well, I would say.

Code: ● ERAS Nurse > OP Prep Gewicht: 0

IG Position: 27 - 27

I

Did you prepare for the surgery differently through these things in the preoperative consultation than you would have otherwise done?

B

Yes, I think so, as I said, the time. I just had more time to familiarize myself with everything. Let's call it like that.

Code: ● ERAS Nurse > OP Prep Gewicht: 0

IG Position: 20 - 22

I

Alright. From your perspective, were you able to distinguish who actually belongs to which profession?

B

Yes yes, of course.

Code: ● Interdisciplinarity > Knowing Team members Gewicht: 0

IG Position: 132 - 134

I

Okay, wonderful. To what extent were your relatives involved in your treatment process, yes, included?

B

Yes, my partner was involved, but I didn't bring her to the preparation discussion. (I: Okay.) And that was a disadvantage, so it's better if the, well, the partner comes along. I would do it differently today, back then I thought to myself: "Yeah, a test and a bit of info." Maybe in the future, you can point out more that it makes sense for the life partner to be there. (laughs)

Code: ● Involvement of relatives Gewicht: 0

IG Position: 107 - 109

I

Yes, alright. Would you still say, though, that perhaps afterwards - especially during the time in the hospital - your relatives received sufficient information?

B

Yes, my partner just says that the surgery was apparently, uh, finished by 11:30 a.m. and she was called at 1:00 p.m. - I think those one and a half hours of worry could have been spared if they had told her.

Code: ● Involvement of relatives Gewicht: 0

IG Position: 111 - 113

I

Yes, absolutely yes. Overall, then in the process, maybe even afterwards, did you feel that you were also involved? So especially in your case, your partner.

B

Yes, since she was often on-site, she was at the hotel very close to the hospital, um, so she was quite involved, yes.

Code: ● Involvement of relatives Gewicht: 0

IG Position: 115 - 117

Well, at the beginning, you have to sort things out again. I had my wife with me, because four ears always hear more than two. And I think that was quite good, um, just then - yes, at the beginning it was a lot on that day.

Code: ● Involvement of relatives Gewicht: 0

IG Position: 34 - 34

I

Yes, okay. And did you also, apart from that, for example, gather information or have further conversations beforehand?

B

Well, with medical personnel, I would say no. But of course, within the family - of course, we talked about various things.

Code: ● Involvement of relatives Gewicht: 0

IG Position: 40 - 42

I

Alright. Then, to what extent were your relatives involved in your treatment process?

B

Yes, my wife was even present at the information meeting at the end of March. She was also there at the first meeting in February, where the ERAS concept was presented to me. My wife also sought contact with the woman from psychosomatics, and that worked won-derfully, so in the end, she was neatly integrated. And otherwise, of course, my family also received information from me that I had received before, yes - but they were involved.

Code: ● Involvement of relatives Gewicht: 0

IG Position: 104 - 106

I

Alright. Can you also determine which professional group or which professionals contributed to your relatives - especially your wife - being so well involved?

B

Well, mainly from ERAS - of course. Yes, and otherwise, I would say - my wife received a call after the surgery, from the doctor - it was quite short - but so far, that everything is okay, yes. That was another piece of information that was conveyed, and otherwise, I don't know now - my wife spoke less with the ward staff. So that, that went through me because I was awake again relatively quickly, or, yes - if something was wrong, I naturally discussed it primarily with the people.

Code: ● Involvement of relatives Gewicht: 0

IG Position: 108 - 114

I

hen maybe as a conclusion, if you could break it down somehow, or maybe you have one or two points that immediately come to mind - which measures or things or people would you say have personally helped you the most during the treatment?

B

Well, from a personal standpoint, definitely my family.

Code: ● Involvement of relatives Gewicht: 0

IG Position: 204 - 206

I

Then let's move on to the next topic: To what extent were your relatives involved in your treatment process?

B

Well, especially my sister, with whom I do a lot together, she was quite well involved, yes. And she was also, uh, always informed accordingly and, uh, she also had to struggle quite a bit with the situation. And she was also quite well looked after. Yes, so that was actually a great thing.

I

Okay. How were you able to support your relatives in this process - was there anything?

B

How - um? Just being there, yes.

Code: ● Involvement of relatives Gewicht: 0

IG Position: 128 - 134

I

Yes, okay. Was there again a specific professional group that particularly contributed to the involvement of your sister in your case?

B

That were the ladies up from ERAS [Psychosomatics, ERAS Nurse].

Code: ● Involvement of relatives Gewicht: 0

IG Position: 135 - 138

And that also helps to calm the psyche a bit, I would say, yes. And not just for me, but also for my relatives - primarily for my sister, yes.

Code: ● Involvement of relatives Gewicht: 0

IG Position: 166 - 166

That is also a significant aspect, that as I said, the relatives are accordingly involved there, yes. And I found it pleasant that my sister was involved - and, um, yes, that was just good.

Code: ● Involvement of relatives Gewicht: 0

IG Position: 222 - 222

Well, I have to say, it was really great that my husband could be there too, because in such moments, one is often a bit nervous or forgets to ask about things, and if the partner is there, they can actually step in for you or they have other questions. So I found that very pleasant.

Code: ● Involvement of relatives Gewicht: 0

IG Position: 22 - 22

I

Yes, alright. Then, to what extent were your relatives involved in the treatment process? You mentioned it right at the beginning, with your husband, I believe.

B

Exactly, so I really have to say, just the two weeks before, so it doesn't really matter. My husband anyway, the kids, so I could just pass on information that came firsthand, not something else, yes - that someone told me - but I could definitely pass that on, and - so everyone was really involved.

I

Okay, then in the hospital itself with the information, were your relatives dependent on you to pass on the information? Or were they also informed independently by the staff?

B

Well, I have to say, that worked very well. Because as a relative, you want to know: "Yes, did the surgery go well or is everything working out?" And my husband was actually notified both by the ward and by the doctor about how the surgery went and what happens next. So the communication was really very good.

I

Okay. How were you able to support your relatives in the process?

B

Well, yes, support, yes of course - they naturally give you encouragement, of course. Otherwise, yes, they were, or it was possible at any time for someone to come by, yes.

I

Okay, and when it comes to informing your relatives, did certain professionals, I mean again the various professional groups, particularly contribute?

B

Yes, as I said, my husband then regarding - because there were a few complications during the surgery - then the ERAS ladies called again, who then informed themselves again about what had happened. So he had access to it at any time, too. And as I said, even the surgeon then had a conversation with him. So that was already very pleasant to find out firsthand, yes, how everything went or what happens next.

Code: ● Involvement of relatives Gewicht: 0

IG Position: 92 - 106

Well, the most helpful - is definitely the partner or husband.

Code: ● Involvement of relatives Gewicht: 0

IG Position: 158 - 158

After my wife has been present at the [pre-operative] conversation, she was also actually reassured.

Code: ● Involvement of relatives Gewicht: 0

IG Position: 71 - 71

That was very helpful [the preoperative counselling]. Both for me and for my wife. I was really glad about that. For my wife, it was simply important to get the information first-hand. And when she has a question, that it will also be answered. Well, it helped her in any case.

Code: ● Involvement of relatives Gewicht: 0

IG Position: 22 - 22

You mentioned earlier that your wife was also present at the preoperative discussion. (B: Yes.) Do you know how it was for your wife while you were being operated on?

B

She was just as, well, excited about the discussion beforehand. She was also very glad to have the opportunity to be cared for, to be prepared, yes. So that was also a very positive experience for her.

Code: ● Involvement of relatives Gewicht: 0

IG Position: 69 - 71

I

Okay. Were you alone at this discussion or did you have a companion?

B

No, my wife was with me.

I

Did it help that she was there?

B

Well, it definitely helped her, she could also ask questions there. I don't think it made such a big difference for me.

Code: ● Involvement of relatives Gewicht: 0

IG Position: 32 - 38

She [the wife] really wanted to get the information firsthand. And if she came up with a question, she wanted it to be answered, yes, I'm not really sure.

Code: ● Involvement of relatives Gewicht: 0

IG Position: 90 - 90

B

My son was there.

I

Did it help that he was also there and also got the information?

B

Yes, so we talked about it afterwards, about the topic. That wasn't bad.

I

So would you do it the same way again?

B

Yes, I would definitely take him with me again.

Code: ● Involvement of relatives Gewicht: 0

IG Position: 36 - 44

I

Um. How did your relatives fare while you were in the hospital? Was it advantageous for them to also be present at the preoperative discussion?

B

Well, I didn't really involve my people that much in that. And that was actually okay. I live two hundred kilometers away, so they didn't come to the hospital either. I said they don't have to come to the hospital until they do a COVID test to get in and yeah. So they didn't come at all.

Code: ● Involvement of relatives Gewicht: 0

IG Position: 90 - 92

I

Do you know how your relatives fared in your care and if everything was fine for them?

B

As far as I know, they were informed very quickly after the surgery. And I could also bring my relatives to the preoperative discussion. They were also there. And therefore they were well prepared.

Code: ● Involvement of relatives   Gewicht: 0

IG   Position: 58 - 60

I

Do you know how your relatives were doing during your care? Even if there were advantages or disadvantages due to your care model - do you know that?

B

Yes, well, my husband has taken it relatively relaxed. Or rather, he is simply pragmatic. But my mother was much more nervous than I was before the surgery but that had nothing to do with the care format. That would have been the case anyway.

Code: ● Involvement of relatives   Gewicht: 0

IG   Position: 60 - 63

I

Yes. And, um, if you summarize that now, or look back on it, what is your attitude now, after the whole process and at this point in time?

B

Good, positive. So I can only endorse that, the way it's done is already, um, correct, yes.

Code: ● Involvement of relatives > Yellow   Gewicht: 0

IG   Position: 163 - 165

I

Alright, yes. You mentioned it at the beginning - it's about the rehab stay: How did you generally experience this rehab stay?

B

The rehab stay was good. However, it was a tough, rocky road. Upon my arrival, I was struggling both psychologically and physically. However, as time passed, I gradually acclimated to the various sports, activities, and exercises. It took approximately three to four weeks before I felt truly comfortable in rehab. I do not wish to assign blame to anyone. It is important to note that my delayed comfort was not due to any shortcomings on the part of the rehab facility, but rather a result of my condition.

Code: ● Rehab   Gewicht: 0

IG   Position: 167 - 173

During my second stay, there were some inconsistencies regarding my transfer back to rehab. Initially, I was told that I would stay in the university hospital until I was completely fit again. However, the next day, other assistant doctors suggested that I should go back to rehab the following day. I contacted the senior physician to clarify the situation. Yes, it was a reasonable conversation and the solution was for me to stay one more day. I later discovered that it was also important for me to return to rehab at that time, as not doing so would have hindered my progress. The continuous interruptions caused some bureaucratic deadlines, but this is to be expected.

Code: ● Rehab   Gewicht: 0

IG   Position: 82 - 82

I

Then let's also move on to your rehab stay: How did you overall experience that?

B

The rehab stay was, um, ultimately just as expected - it was okay. The rehab lasted a total of four weeks, yes. And then you just noticed how it was going uphill bit by bit, and I felt that was exactly what I had expected - and that was pleasant. But at some point, after four weeks of rehab, you slowly want to go home again.

I

Yes, absolutely, I can relate to that very well. I assume you went to rehab relatively early after your surgery, right?

B

That was directly, um, oh, what can I say, after the complications were taken care of, um, to a certain extent. (I: Yes.) - and that was okay. Then I went directly to rehab right after the hospital stay - I didn't go home first.

I

How did you feel about - or how did you feel about the fact that you went directly into this rehab?

B

Well, I found that very pleasant. It's understandable if other people say, "Now I just want to be home for a bit." So maybe one or two nights at home - I wouldn't have considered that as bad, but to get directly and quickly into rehab, that's very important, in my opinion.

I

Yes, I can certainly see that it might be generally positive, as you just mentioned, to be at home first. But did you feel that through this, yes, early rehab, you were in some way well taken care of - especially concerning any concerns after such complications?

B

Well, from my point of view, it's very good to go directly or as quickly as possible into rehab, yes. Because if you first get out of the hospital after such a surgery and then, I don't know, spend two, three weeks twiddling your thumbs. That would have scratched my psyche quite a bit - before going to rehab. So then you just have more or less continuous care right away, and I found that very pleasant.

Code: ● Rehab Gewicht: 0

IG Position: 188 - 202

I

Then when we look at your health status after rehab: How fit were you upon discharge from rehab?

B

I felt - compared to how the situation was overall – that I was in good shape after rehab, yes. I'm still nowhere near one hundred percent, but I'm already doing well, yes. So, as I said, I am fit enough that

you can cope with everyday life and that I can do everything independently at home again. So the rehab has put me back on my feet.

Code: ● Rehab Gewicht: 0

IG Position: 204 – 206

The rehab was positive throughout. I must say, I was practically on rehab after four days. At that moment], it was the case that I thought to myself, "Oh my God, this is all very much." And I couldn't do anything - but after two to three days - but then after two or three days of rest and getting used to it, and when everything slowly started with smaller training sessions I saw that compared to other patients, yes, I'm actually relatively far along with the recovery process already.

Code: ● Rehab Gewicht: 0

IG Position: 134 - 135

I

Alright, yes. Then the next question is about the rehab stay. You've already started on that. Um, maybe first of all: How did you feel about being admitted to a rehab facility so quickly and directly?

B

So that was really great for me and, first and foremost, for my husband and the other rela-tives. It was ideal because I have to be honest, in the first few days in rehab, there were often situations, yes, where you suddenly have heart palpitations or a situation where you think, "Wow, is that normal now?". So, if I had been at home, I would have been worse able to deal with it. And in fact, so would my husband, because in that case, he doesn't know what to do either. Above all, he's not available the whole day, he's out of the house and at work. So I think it was even better for him because he had a calm feeling that I was in good hands. And I felt the same way, I would not have been able to cope so well with some situations at home, probably without calling either a doctor or an ambulance. So the uncertainty is completely taken away from you.

I

Alright. Besides this feeling of security, were there any other things in rehab that supported your recovery?

B

Definitely. There, of course, primarily the doctors - as well as the nursing care. Then I have to say what was really very pleasant - because through the whole surgery, the upper body, the back, everything is tense, it hurts from the posture - and there it naturally helps right away when you already have physiotherapy, a massage, or heat treatment from the second day. That besides the heart surgery, it actually provides relief.

I

Yes, so how would you describe that then - you already mentioned that the rehab stay was very positive. How fit were you then upon discharge from rehab?

B

Well, I was actually, so after four weeks - because I had a one-week extension -, I was really very good. So I could take walks, actually ride the stationary bike as well. In terms of lung capacity, I was actually, yes, quite fit, I have to say.

Code: ● Rehab Gewicht: 0

IG Position: 140 - 150

I didn't find that so great in rehab, yes. Because in the first week I could hardly do anything. It was perhaps also poorly timed, the period when I was in rehab was exactly over Easter. Nothing happens in rehab anyway during Easter, yes. In terms of the treatments, it was a bit, the first week was a bit disappointing, I would say. That could have been scheduled a bit better.

Code: ● Rehab Gewicht: 0

IG Position: 55 - 55

So and then my therapy, my rehab actually only really started in the second week. But I couldn't do much [except for cycling at the ergometer] then because I couldn't put any weight on my sternum. Yes, so everything was a bit more difficult. The medical care at the rehab was great. (I: Yes.) Yes. The care itself, in rehab, the medical care, was great. It was phenomenal, I have to say. But the treatments, you can really think about that. What's done there, I can do in my garden as well.

Code: ● Rehab Gewicht: 0

IG Position: 59 - 59

So the medical care was, as I said, great in rehab. But the therapeutic measures I had, I couldn't do some of them at all. And overall, it was too little, yes.

Code: ● Rehab Gewicht: 0

IG Position: 104 - 104

After surgery, patients may experience difficulty performing certain activities during rehabilitation due to chest pain. Other exercises that involve the upper body and chest area may be challenging due to pain. However, stationary biking and walking are still possible. My muscle building and recovery only occurred after the official rehab. I participated in an additional program which improved the recovery process.

Code: ● Rehab Gewicht: 0

IG Position: 116 - 118

But in itself, the physiotherapy was very good. And what I also appreciated, which I noticed compared to others, was that they conducted their sessions in groups of fourteen, fifteen people, and we were only four. That allowed us to benefit much more from the sessions. At least in my perception, it was a completely different way of working. With exercises, tasks, explanations.

It was more individualized, yes. (I: Okay, great.). It was like when we were outside, for example, with the therapist and four people, where everyone could walk, he always had everyone in sight and everyone could walk as well as they could. He was with the weakest, but I always felt taken care of. Even though I was walking alone.

Code: ● Rehab Gewicht: 0

IG Position: 62 - 63

I

Would you have liked to go home between the hospital and rehab?

B

No, for me, it was good to continue right away there. I think if I had gone home for a few days or maybe longer, I wouldn't have known what to do. That would have been a break. It's just a different environment, so the process that had already started in the hospital continued directly. So I was glad that it continued right away. Of course, it's also a long time being away from home. I was away for almost five weeks. But I would do it like that for myself, for my perception, anytime again.

Code: ● Rehab Gewicht: 0

IG Position: 97 - 99

When I was discharged? (I: Yes.) Well, I always felt relatively fit. And of course, I also had some low points. But the activity never really exhausted me. It was always manageable.

Code: ● Rehab Gewicht: 0

IG Position: 103 - 103

Let's put it this way, rehab does make a difference. There were a few things that weren't optimal for me, but overall, it was really great.

Code: ● Rehab Gewicht: 0

IG Position: 157 - 157

B

Yes, so rehab directly after the hospital definitely makes sense.

I

But if, for example, you had gone home first from the hospital and then come to rehab after two weeks. How would you have felt about that?

B

I would say it's better the way I had it. Because when you then go away from home again -. Immediately afterward, then it's done. I think.

Code: ● Rehab Gewicht: 0

IG Position: 165 - 169

So I was able to do everything at the rehab from the beginning without any problems. So well, the preparation for the surgery was very good. The only thing that didn't sit well with me was that whole day without eating and drinking and without medication. That didn't sit well with me.

Code: ● Rehab Gewicht: 0

IG Position: 50 - 51

I

What you've already mentioned is that you were able to go directly from the hospital to rehab, did you find it good that you were transferred there directly?

B

I found that very good. Because, as I said, because one doctor even thought they could send me home on Monday for that one day. When I thought, oh God, how am I supposed to manage that at home. So for example, I was glad that I always had a bed there where I could raise the headboard.

Otherwise, at the beginning, I wouldn't have been able to get out of bed alone because it hurt so much. And just having this help and also knowing that I'm going to rehab, where there are still doctors and a nurse and somehow if something completely goes off track. There's someone who at least knows what they could do. If I had been at home now. I think that would have worried me a lot. For me, it was really good. Of course, I could do relatively little at the beginning. But I was fine with that because I can train for myself, so to speak. I don't need much support for that now.

Code: ● Rehab Gewicht: 0

IG Position: 93 - 95

I

Could you have imagined instead going home first?

B

Oh no, that wouldn't have been so good.

I

And why?

B

Well, they took care of me there. I was well taken care of. And had to do sports, and if I had something, for example, I had severe back pain from the surgery. And then they took care of it right away in rehab. And after a few weeks of rehab, I almost felt healthy again. They took care of things right away when you said something, they did it right away. Some nerves were pinched after the surgery, and they immediately helped me. That wouldn't have been so good alone at home. I felt safe in rehab.

Code: ● Rehab Gewicht: 0

IG Position: 93 – 99

I

How was rehab for you?

B

Well, it was good there. The beginning was a bit weird, but then it was fine. Yes, on the first day, they immediately let me walk around a lot, back and forth and all around, there in the rehab center. It was a bit much, really a bit much, but well, they apologized afterwards, they hadn't even realized that I had just been operated on, apparently.

Code: ● Rehab Gewicht: 0

IG Position: 53 - 55

I

How fit were you then upon discharge - since you mentioned, um, before rehab, or when you started rehab, it was really very bad and that for a longer time - upon discharge, what was it like?

B

Um, I was much better, well, also, also in terms of fitness - there's just no comparison. As I said, on the first day, I could hardly get up, they wheeled me to the room in a wheelchair, and at the end of rehab, um, I was regularly doing my stationary bike training, so it was really a big leap - an improvement.

Code: ● Rehab > Daily life afterwards Gewicht: 0

IG Position: 179 - 181

I

I Okay. And how fit would you say you actually were upon discharge from the inpatient rehab?

B

Well, how I was feeling? I wasn't fit after the rehab. I noticed that relatively quickly, on the day of discharge, or the day after - colloquially, I would have said, it pulled the plug on me in the afternoon. Despite lying down for several hours, I couldn't recover that day. So, I would say I was still below 50 percent. The patient's performance has improved significantly since the surgery, but it is still below their normal level. They are able to manage a day reasonably well, but there is still room for improvement. It is important to note that the rehabilitation centre provides various treatments throughout the day, which may not be available at home. It is important to note that the rehabilitation centre provides various treatments throughout the day, which may not be available at home. However, they experienced difficulty in doing so. It is important to note that the rehabilitation centre provides various treatments throughout the day, which may not be available at home. Additionally, the patient had some office work to catch up on after being away for five weeks, which they wanted to complete quickly. Those were the issues, and then some physical activity ensued, leading to a complete halt.Code: ● Rehab > Daily life afterwards Gewicht: 0

IG Position: 192 - 194

So from that perspective, I think it's a good thing, and yeah, many people say that too. Even my family doctor says, well, after about five weeks, when I first saw him, things were already looking good, he said. As I said, if you go back a few years, someone would never have stood like I did after five weeks.

Code: ● Rehab > Daily life afterwards Gewicht: 0

IG Position: 198 - 198

Last week, we walked quite a distance - you're from Augsburg too, I suppose. (I: Yes, I moved here.) Um, so from the South Sports Center in Haunstetten to the square and back. (I: Yes.) Um, where I already noticed, that's enough now. So - a normal, healthy person, they probably wouldn't stress about walking there, yes. But for me, it became quite tedious towards the end. But that was actually the furthest I've managed to walk so far. According to my pedometer, it was 12,000 steps or so, a little more. I don't think that's bad, yes. But as I said, I naturally thought I could do that right after discharge from rehab - to walk such distances, and that - that definitely wouldn't have been possible. (I: Okay.) But I can already feel that it's still getting better - even the pain is improving somewhat. So they're still there in the upper area, but that's also getting a bit better, and I think - as everyone has said: "Patience, patience, it takes weeks." (I: Yes.) And I just have to muster that now.

Code: ● Rehab > Daily life afterwards Gewicht: 0

IG Position: 202 - 202

As I was discharged? (I: Yes.) Well, I always felt relatively fit. And of course, I also had some low points. But the activity never really pushed me to the edge of exhaustion. It was always manageable.

Code: ● Rehab > Daily life afterwards Gewicht: 0

IG Position: 103 - 103

I

Yes, but was there still something during your rehab stay that really helped you, so that ultimately, after a certain time, you could feel comfortable?

B

What helped was that I gradually started feeling better, yes. So at the beginning, I really, uh, what do I know - I struggled with my lungs, I had - well, with breathing, I got some cardiac arrhythmias at night - it was all a difficult time. But I can't blame rehab for that, because there was always someone there taking care of me - that was the most important thing, I believe. I already felt safe in rehab.

Code: ● Rehba > Intensity Gewicht: 0

IG Position: 175 - 177

I

Alright. (B: Yes.) Then let's move on to your rehab stay - you briefly mentioned it earlier, maybe a bit more detailed - how did you feel about the fact that you came to rehab so early?

B

On the one hand, I was happy to get out of the hospital as soon as possible. Naturally, there was the expectation that if I'm in rehab, then things will improve very quickly, right? (I: Yes.) But that wasn't the case, yeah, but it was very bumpy initially.. In the end I came to rehab on a Wednesday, and on Thursday, the first treatments started, but in the evening, I already had a fever, yes - so those were the first signs that something wasn't quite right. And on Friday, they more or less took me out of commission and prescribed rest, and then the first examinations were also done - ultrasound. And then I actually had contact with several doctors who ultimately approached me with the request to say that I should go on monitoring. (I: Yes.) Because they didn't have a good feeling, and I also noticed for myself that I was actually feeling worse from hour to hour, yes. (I: Okay) And that was practically, in the end, the beginning of the end, of the first rehab stay - a few hours later I was back in Augsburg. And um, when I came back, on the following Wednesday, of course, there wasn't much going on that Wednesday either. They didn't know when I was coming, and on Thursday, the program practically started again - although I also noticed that they had already started with a gentle program at the beginning. Because they naturally wanted to observe how I was actually doing and how I was coping. (I: Yes.) Not that there's a relapse, I think that was absolutely okay like that. Um, so it actually took some time until I really, yes, how should I say, really arrived there. Yes, because on the next day, after the second arrival, my temperature rose again - not, I mean, it wasn't a fever, but it was an elevated temperature. And then, of course, that was an alarm for me, and it wasn't until the following week on Tuesday, so it was after this first May holiday, that there was another ultrasound examination, which then confirmed again that nothing had formed - everything was okay. And then I actually mentally arrived a bit more for myself, but of course, I noticed that the body's resilience is limited. So if I demanded too much from myself, and it could just be a walk - on that huge grounds there in Höhenried - um, if that was too much, then that was it for the day, yes. (I: Yes.) That happened to me a few times because I couldn't assess it for myself - you have certain treatments, then there's also downtime in between, then you think: "Well, then you'll go for a walk, it'll do you good - oxygen, movement." Um yeah, and sometimes it was too much of a good thing. And um, then it was about - because I had lost five days, or actually it was six days - for me, it was also about the topic of extension. The first extension went relatively smoothly, but there wasn't a second one anymore because, yeah, according to the statement, unfortunately, there were no beds available anymore. (I: Okay.) That was of course a pity, in the end, because I just noticed that the last week of rehab for me was the most beneficial. Probably always like that, yeah, that it's most

beneficial towards the end, but it would have been even nicer if I had another week because I believe that would have helped even more.

I

I understand.

B

We have an alternative program for that now, it was a suggestion from the attending physician who said that it's a follow-up rehab - and I've been doing that since last week. (I: Okay.) Just this morning again. (I: Yes.) Um, it's quite close to the hospital, and I don't find that bad now, yes, that I can have an additional training session for myself, where I can do a bit more strength training. And I don't think that hurts now - there are 24 sessions that I can use. And that's certainly - it's not a replacement for inpatient rehab, but it's better than not having it, yes.

I

Yes, I believe that. And overall - I would just limit it more or less to your stay in rehab, after you arrived there for the second time (B: Yes.) - would you say that it ultimately contributed to your recovery?

B

Definitely.

Code: ● Rehab > Intensity Gewicht: 0
IG Position: 180 - 191

I

Would you have liked to go home between the hospital and rehab?

B

For me, it was so good to continue right away. If I had been at home for a few days or maybe longer, I wouldn't have known what to do. That would have been a break. It's just a different environment and so the process that had already started in the hospital continued directly. And so it went right on in the process that had already started in the hospital. Of course, it's also a long time to be away from home. I was away for almost five weeks. But I would do it like that for myself, for my feeling, anytime again.

Code: ● Rehab > Point of time Gewicht: 0
IG Position: 97 - 99
