# Supplementary figures and images for: Implementation of an innovative ERAS protocol in cardiac surgery: A qualitative evaluation from patients’ perspective
Source: PLoS One. 2024 May 10;19(5):e0303399. doi: 10.1371/journal.pone.0303399 (PMC11086837; doi:10.1371/journal.pone.0303399)

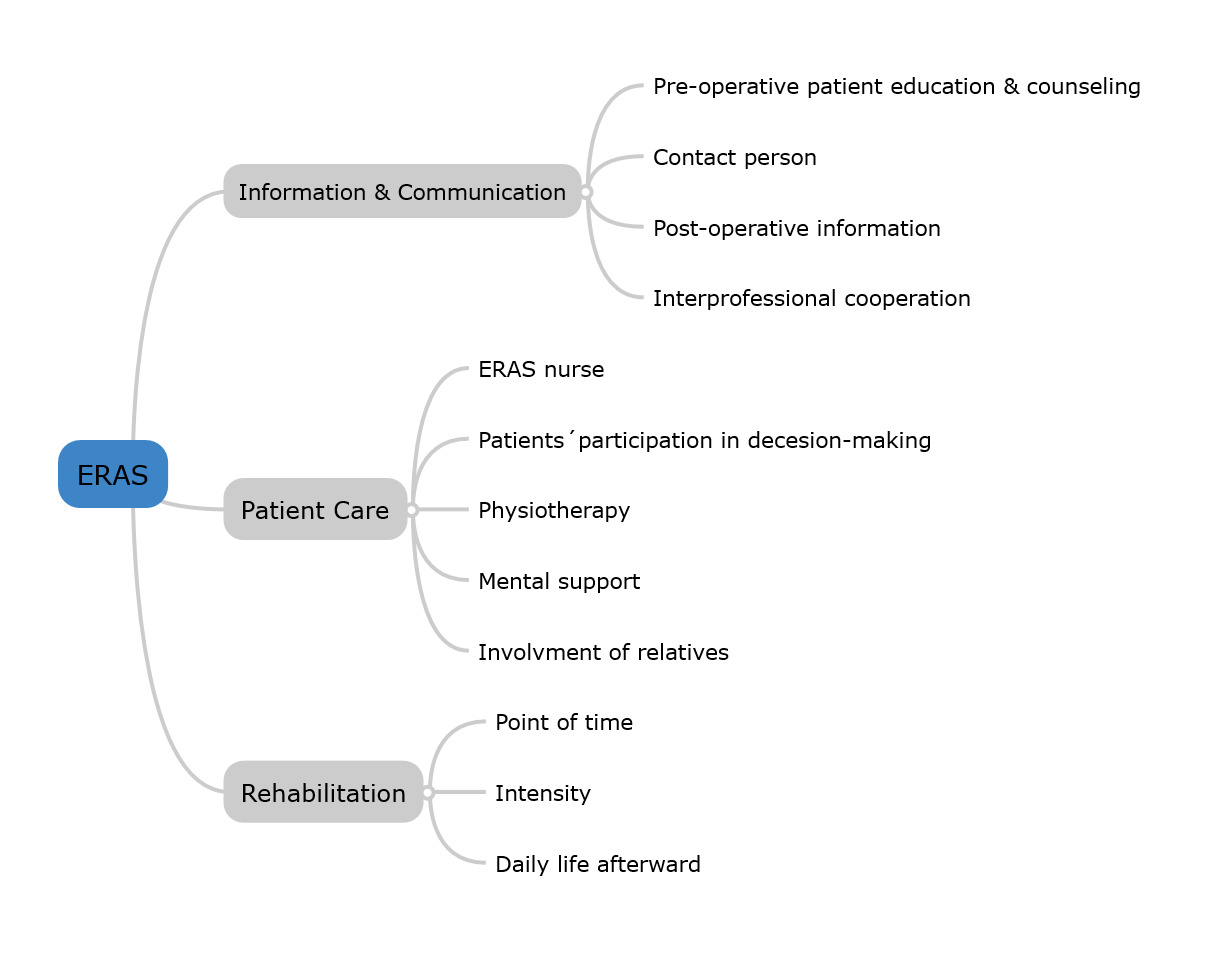

Supplement: S1 Fig — (TIF) [file pone.0303399.s002.tif]
